# Active methylotrophic methanogenesis by a microbial consortium enriched from a terrestrial meteorite impact crater

**DOI:** 10.1128/mbio.03017-25

**Published:** 2025-11-25

**Authors:** Femke van Dam, George Westmeijer, Maryam Rezaei Somee, Marcelo Ketzer, Riikka Kietäväinen, Shuhei Ono, Stefan Bertilsson, Jennifer C. McIntosh, Mark Dopson, Henrik Drake

**Affiliations:** 1 Centre for the Environment (CENWIN), Linnaeus University 4180, Kalmar, Sweden; 2 Centre for Ecology and Evolution in Microbial model Systems (EEMiS), Linnaeus University 4180, Kalmar, Sweden; 3 Department of Chemistry, Umeå University 201287, , Umeå, Sweden; 4 Department of Aquatic Sciences and Assessment, Swedish University of Agricultural Sciences 8095, Uppsala, Sweden; 5 Geological Survey of Finland 52926, Espoo, Finland; 6 Department of Geosciences and Geography, University of Helsinki 205533, Helsinki, Finland; 7 Department of Earth, Atmospheric, and Planetary Sciences, Massachusetts Institute of Technology 517829, Cambridge, Massachusetts, USA; 8 Department of Aquatic Sciences and Assessment, Swedish University of Agricultural Sciences and Science for Life Laboratory 166469, Uppsala, Sweden; 9 Department of Hydrology and Atmospheric Sciences, The University of Arizona 8041, Tucson, Arizona, USA; Georgia Institute of Technology, Atlanta, Georgia, USA

**Keywords:** methanogenesis, Archaea, metagenomics, metatranscriptomics, methylotrophy

## Abstract

Microbial methane generation (methanogenesis) is an important metabolic process in the terrestrial deep biosphere and is an analog to early Earth as it is proposed to be one of the most ancient metabolisms on Earth. Signs of methanogenesis in meteorite impact craters are of particular interest in this respect as these settings are proposed hot spots for deep microbial colonization of the upper crust. Yet, reports of active deep rock-hosted methanogenesis are scarce, particularly for methylotrophic methanogenesis, while reports from terrestrial meteorite impact craters are completely lacking. Here, we used indigenous communities in cultures enriched from 400-m deep fluids to confirm and characterize active methane production from several carbon donors, including indigenous oil, in a terrestrial impact crater at Siljan, Sweden. Metagenomic and metatranscriptomic data of the methane-producing cultures revealed a consortium dominated by *Acetobacterium* sp. KB-1 and *Candidatus* Methanogranum gryphiswaldense, mediating methanogenesis solely via the methyl-reduction pathway, and resulting in a δ^13^C_methanol-methane_ isotope enrichment of up to 98.6‰. These results provide insights into methylotrophic methanogenesis in deep subsurface environments in general, and in particular in fractured meteorite impact structures.

**IMPORTANCE:**

This study revealed that microbes enriched from groundwater in a 380-m deep borehole within the Siljan meteorite impact crater in Sweden were capable of producing methane, a key greenhouse gas. This is especially significant because it is the first proof of active methanogens in an impact crater and showing a specific pathway of methane production—methylotrophic methanogenesis—is present in the deep terrestrial subsurface, an environment that is typically hard to study. These findings shed light on life in extreme conditions on Earth and show that meteorite craters can be biological hotspots, rich with ancient life processes.

## INTRODUCTION

Methanogenesis is one of the oldest known metabolisms on Earth (1) and was influential in governing the Earth’s early atmosphere (2). Based on molecular clock analyses (3) and methane-bearing fluid inclusions (4), methanogenesis is estimated to have evolved more than 3.5 Gyr ago, when the Earth’s atmosphere was nearly absent of free oxygen, the sun was much dimmer, and temperatures were higher owing to a strong greenhouse gas effect caused by high methane concentrations (5).

Meteorite impact craters are of particular interest as they are proposed to be hot spots for life’s onset of colonization on barren planetary bodies (6). This is because the impact creates heat, fluid circulation, and redox gradients that make nutrients and redox couples available while also increasing the porosity and permeability of rocks to create pore space for deep microbial colonization (7, 8). Reports of ancient isotopic and morphological biosignatures in terrestrial impact craters confirm that the fractured bedrock is prone to microbial colonization (9–11). Yet, studies of deep microbial communities in terrestrial impact craters are surprisingly few and have to date not been focused on methanogenesis. Cockell et al. (12) reported microbial abundances in the Chesapeake Bay impact structure, confirming the presence of microbial life in the fractured bedrock. Furthermore, Quraish et al. (13) extracted DNA from fractured granites and mineral veins from an 829 m-long core within the Chicxulub impact crater and reported on the different microbial communities between granitic and impact-altered suevite, melt rocks, and serpentinized ultramafics.

**Fig 1:**
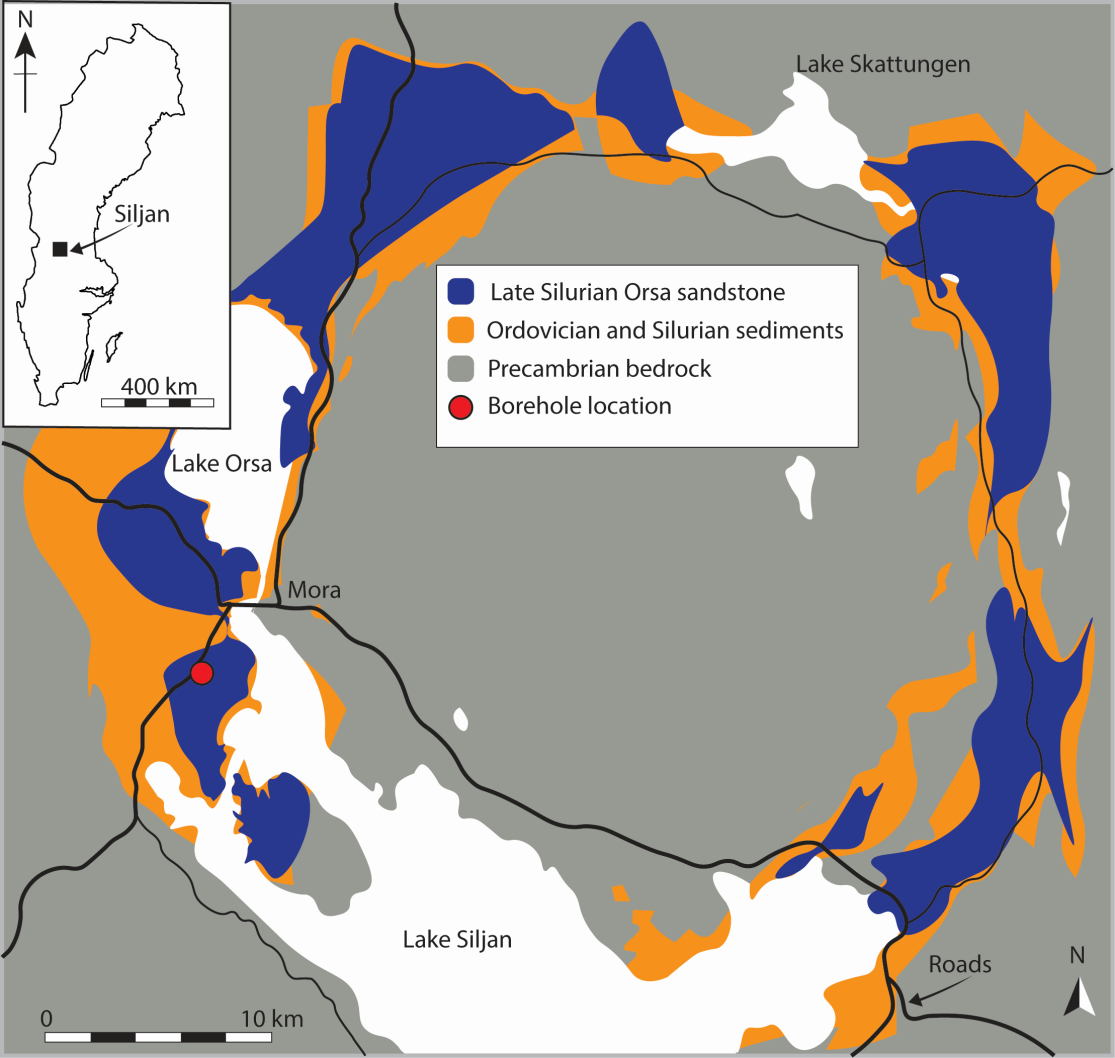
Map depicting the location of the sampled borehole in the Siljan impact structure, central Sweden. The borehole is located at Lat 60.98° N, Lon 14.52° E, in the sedimentary rim of the impact structure. Figure modified after (14).

The 380.9 ± 4.6 million-year-old (14) Devonian Siljan impact structure in central Sweden is of particular interest as seeping hydrocarbons, including methane from deep fracture networks, have been known for hundreds of years (15) (Fig. 1). In the crater rim depression, Ordovician and Silurian sedimentary rocks exist as downfaulted successions of up to 500-m depth and overlay the Paleoproterozoic granitoid bedrock that crops out in the central dome and surrounding areas (16). Thermogenic hydrocarbon gases, seep oil, and bitumen are interpreted to originate from organic-rich shales occurring in the ring depression (17, 18). Isotopic, morphological, and organic geochemical biosignatures for ancient microbial methanogenesis are reported from fracture coatings in both the sedimentary successions and the basement rocks (18). Accumulations of methane have been found in this structure, including multiple lines of evidence for ancient methanogenesis dating back to 80 ± 5 million years ago (18, 19) and the presence of methanogens in the deep aquifer (20). The environmental groundwater had a 16S rRNA gene amplicon-based community dominated by Minisyncoccota (previously Patescibacteria/CPR clade) but also with a low abundance of hydrogenotrophic, acetoclastic, and methylotrophic methanogens, such as the methylotrophic methanogen *Methanomethylophilaceae* UBA71, as well as acetogens *Acetobacterium* sp003260995 (KB-1) and *Acetobacterium dehalogenans* (20). However, due to the low biomass in the deep biosphere and low abundance of methanogens in particular, the metabolic pathways of methanogenesis by the indigenous communities remain unknown.

Here, we present metagenomic and metatranscriptomic data of heterotrophic, methanogenic enrichment cultures from microbial communities in a 380-m deep borehole in the Siljan impact crater. This was complemented with a time series of the evolution of CH_4_ concentration, δ^13^C_CO2_, δ^13^C_CH4_, and δD_CH4_, as well as multiply substituted isotopologues of methane. The objective was to decipher previously unexplored active microbial methanogenic pathways in a terrestrial impact crater.

## RESULTS AND DISCUSSION

### Enrichment of methanogens and methane production

The groundwater used for microbial incubations was pumped from a 400-m deep borehole at the contact between the sedimentary and basement units in the crater rim area. Based on low Cl^−^ concentrations (1100 mg L^−1^) and δD_H2O_ and δ^18^O_H2O_ values of −87.9‰ and −12.4‰, respectively, the groundwater was likely formation water modified by meteoric water incursion. The groundwater sulfate concentration is relatively low (20 mg L^−1^) with high alkalinity (713 mg L^−1^ CaCO_3_), a pH of 6.86 and a temperature of 14.3°C (20).

Anaerobic enrichment cultures were prepared to select for heterotrophic methanogenesis using either enrichment media (treatment M) containing the substrates acetate, methanol, casein, and yeast extract; enrichment media supplemented with oil (treatment MO), or enrichment media without any electron donor supplemented with oil (Table S1). The oil collected at Siljan has a strong aliphatic character, with 68.1 wt% saturates, 23.1 wt% aromatics, 8.4 wt% polar compounds, and 0.4 wt% asphaltenes and indications of biodegradation (20). Post-hoc comparisons with the control showed the methane concentrations in treatment M differed significantly starting day 113 (*P* < 0.001) in the first experiment, with a maximum methane concentration of 14,969 ± 4,712 ppm (*P* < 0.001; *n* = 4) for treatment M after 169 days, while treatment MO reached a maximum methane concentration of 3,913 ± 3,309 ppm (*n* = 4). The high standard deviation was due to two replicates producing only 737 ± 67 ppm, which did not differ from control (*P* > 0.9), whereas the others produced 7,088 ± 1,521 ppm (*P* < 0.001; *n* = 2). The culture without any electron donor did not produce significant amounts of methane and did not differ from control at any timepoint (maximum 5.3 ± 0.1 ppm, *n* = 4; Data S1; Fig. S1).

**Fig 2:**
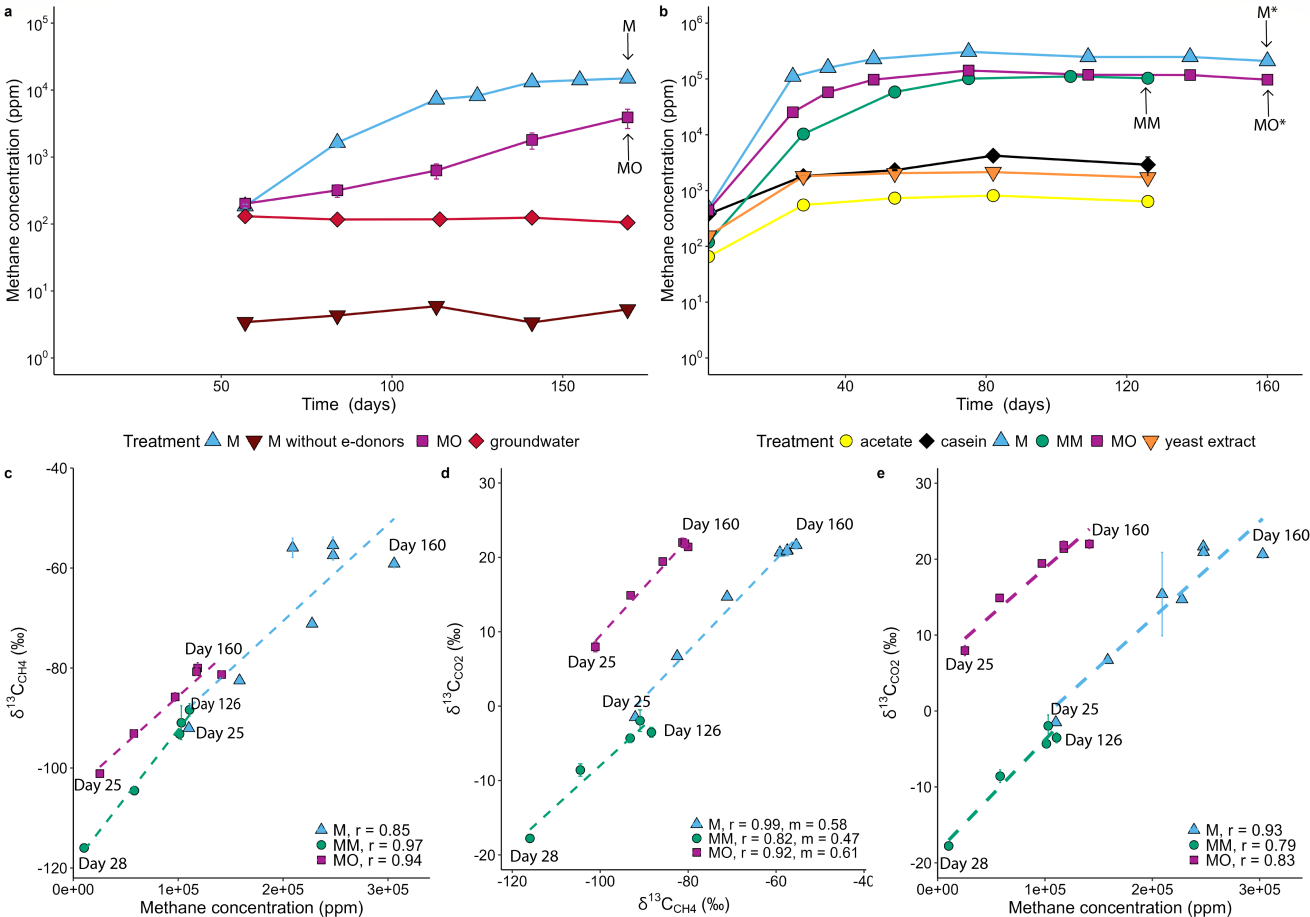
Concentrations and isotopic data from headspace gases of the incubation experiments. (**a**) Development of CH_4_ concentrations in the initial incubation, first measurement at day 57 with a concentration of 100 to 300 ppm; (**b**) development of CH_4_ concentrations in the second incubation, first measurement after 1 day with a starting concentration of 100 to 600 ppm; (**c**) δ^13^C_CH4_ vs CH_4_ concentrations, second incubation; (**d**) δ^13^C_CH4_ vs δ^13^C_CO2_, second incubation; and (e) evolution of δ^13^C_CO2_ vs CH_4_ concentrations, second incubation. Pearson correlation is shown as stippled lines and r values in legends of panels b–d. Number of replicates (*n* = 4) and standard deviations are represented in the plot as error bars. Arrows indicate the point of DNA/RNA sampling, and *denotes the difference between samples from first and second incubations.

Based upon the methane production measured in the initial enrichment cultures, sub-cultures from established methane-producing cultures M and MO were performed to further enrich for methanogenic communities. These cultures showed the highest increase in methane concentration for the M and MO enrichments, with significant methane production at day 25 (*P* < 0.001 for both; Fig. S2), reaching a maximum of 209,133 ± 34,804 ppm and 96,777 ± 16,102 ppm, respectively (*n* = 4, Fig. 2a), followed by treatments where methanol was included as the sole electron donor at day 75 (*P* < 0.001; treatment MM; 110,819 ± 20,270 ppm, *n* = 4). In contrast, the methane concentration in the enrichment media amended with acetate, yeast extract, and casein as sole electron donor showed an initial, smaller burst of methane, without any further increase and no significant difference from control (Fig. S2). Casein treatment reached a maximum methane concentration of 4,214 ± 1,037 ppm (*n* = 4) at day 82 with no significant methane production (*P* < 0.92; treatment compared to control), and similar results for yeast extract treatments (*P* < 0.99), reaching a maximum of 2,142 ± 69 ppm (*n* = 4), whereas acetate amended cultures reached up to 812 ± 39 ppm (*P* < 1; *n* = 4; Data S2). Non-biological methane production was monitored with filtered groundwater and medium controls during all incubations to ensure potential dissolved methane release from the media or groundwater was accounted for, in which no indication of methane production was detected over the incubation period.

### Gas composition and isotopes derived from the enrichment cultures

As the concentration of CH_4_ in the cultures increased, the carbon isotope signatures of both CH_4_ and CO_2_ became enriched in the heavy ^13^C isotope (Fig. 2b and d). Gradually higher δ^13^C_CH4_ values (starting at −92.0‰ ± 0.7‰, *n* = 4, increasing to −55.9‰ ± 3.8‰ V-PDB) are in line with reservoir effects of a closed system and microbial substrate depletion (21), owing to the preferential incorporation of the light ^12^C isotope in the methane molecule. Initial δ^13^C_CH4_ signatures could not be measured due to low concentrations, but a projection of the regression line (δ^13^C_CH4_ vs CH_4_ concentration; Fig. S3) to the point where the CH_4_ concentration was equal to zero indicated a δ^13^C_CH4_ value of −118.6‰ ± 0.5‰ (*n* = 4) for the MM replicates. This value was interpreted as the initial methane δ^13^C signature prior to the influence of reservoir effects. The methanol δ^13^C value in the substrate media was determined to be −19.4‰, suggesting a methanogenesis-related isotope enrichment (ε^13^C_methanol-methane_) of −98.6‰. The observed isotopic fractionation was in line, albeit slightly higher, with previous reports for ε^13^C_methanol-methane_ of −94‰ to −68‰ (22) and −83.4‰ to −71.6‰ (23). At the time of sampling for biomass and ending the MM incubation, δ^13^C of methane was still increasingly becoming heavier, indicating that methanogenesis was still ongoing.

No clear trend in the CO_2_ concentration was observed, but a stable CO_2_ concentration in the incubations was measured with increasing δ^13^C_CO2_ values resulting from closed system substrate depletion (Fig. 2d; Data S3). The MO incubation δ^13^C_CO2_ values were extremely high (up to 47‰ in the first incubation after 169 days), possibly due to the carbon substrate being initially enriched in the heavy ^13^C isotope, with the oil containing alkane and aromatic carbon isotope ratios of −30.7‰ and −30‰, respectively. The CO_2_-CH_4_ isotope fractionation (Fig. 2c) can inform about the pathway of methanogenesis; however, according to Vinson et al. (24), this apparent fractionation is not a strict substrate-to-product fractionation but can reflect multiple pathways. The δ^13^C_CO2-CH4_ relationship can integrate effects from several ongoing processes: (i) competition for substrates by multiple species and substrate depletion/closed reservoir isotope affecting both metabolic products CH_4_ and CO_2_, with different fractionations; (ii) CO_2_ production as a byproduct of methanogenesis as both heavier CH_4_ and CO_2_ are produced simultaneously; and (iii) bacterial processes occurring separately from methanogenesis, such as sulfate and iron reduction, producing CO_2_ that is isotopically heavier in general compared to CH_4_. In this enrichment culture, methylotrophic methanogenesis does not produce CO_2_ as a byproduct, making point (ii) unlikely. As it was a closed system, process (i) most likely explains why δ^13^C_CO2_ increased in a similar manner as δ^13^C_CH4_. The substrates were becoming increasingly depleted, resulting in ^13^C-enrichment of the substrates due to the reservoir effect, and consequently in the metabolic product.

Methylotrophic methanogenesis is thought to have a larger apparent fractionation of δ^13^C_CO2-CH4_ (>1.058) than acetoclastic methanogenesis, but at values that overlap with hydrogenotrophic methanogenesis (21, 23, 25). The δD_CH4_ value informs on the methanogenic pathways as three of the four hydrogen atoms in methane are derived from the methyl group in acetoclastic and methylotrophic methanogenesis, whereas all four hydrogen atoms are derived from the water molecule in hydrogenotrophic methanogenesis (21). Therefore, methane derived from acetoclastic and methylotrophic methanogenesis has more negative δD_CH4_ values. The δD_CH4_ vs δ^13^C_CH4_ values showed a linear relationship in treatment M but not in MM or MO (Fig. S4). This was an indication of a “reservoir” effect in treatment M, by progressive depletion of the substrate in the light isotope owing to its incorporation in the CH_4_ molecule. The complete headspace gas compositions of the M and MO incubations after 134 days showed no molecular hydrogen gas in the cultures or incubated groundwater (Tables S2 and S3). Incubations carried out on unfiltered groundwater without any added substrate indicated that higher hydrocarbons (C_2_-C_5_) were present in the groundwater. In the headspace of unfiltered water, methane was present with a δ^13^C_CH4_ value of −62.6‰, which is comparable with the field data (18). The δ^13^C_CO2_ value of 6.6‰ was similar again to field data with a value of 6.4‰ (20) but substantially lower than that observed in the M and MO enrichment cultures (up to 22‰ and 24‰, respectively). The presence of ethane and propane in the MO and groundwater samples indicated that these gases were degassed from the oil and were not produced microbially, suggesting a minor thermogenic contribution to the incubation headspace.

Clumped isotopes or multiply substituted isotopologues of methane can be applied as a geothermometer to indicate the methane source and formation process (26). The multiply substituted methane isotopologs were analyzed on day 134, when methane concentrations had sufficiently increased. The clumped isotopes showed a strong disequilibrium, with negative Δ^13^CH_3_D values of −4.14‰ and −5.40‰ for M and MO (Table S4), both being outside of the established geothermometer (Fig. S5). These disequilibrium values are typical for a ‘closed system’ with substrate depletion, such as in an incubation (27, 28). Stolper et al. (29) show that values below −4‰ are only found in laboratory cultures of hydrogenotrophic methanogens and below −5‰ for methylotrophic cultures that were in line with the cultures enriching for methylotrophic methanogens. Taken together, rapid methanogenesis was detected in the enrichments, and a large fractionation was detected for methanol-mediated methanogenesis (ε^13^C_methanol-methane_: −98.6‰).

### Metagenomic and metatranscriptomic sequencing

**Fig 3:**
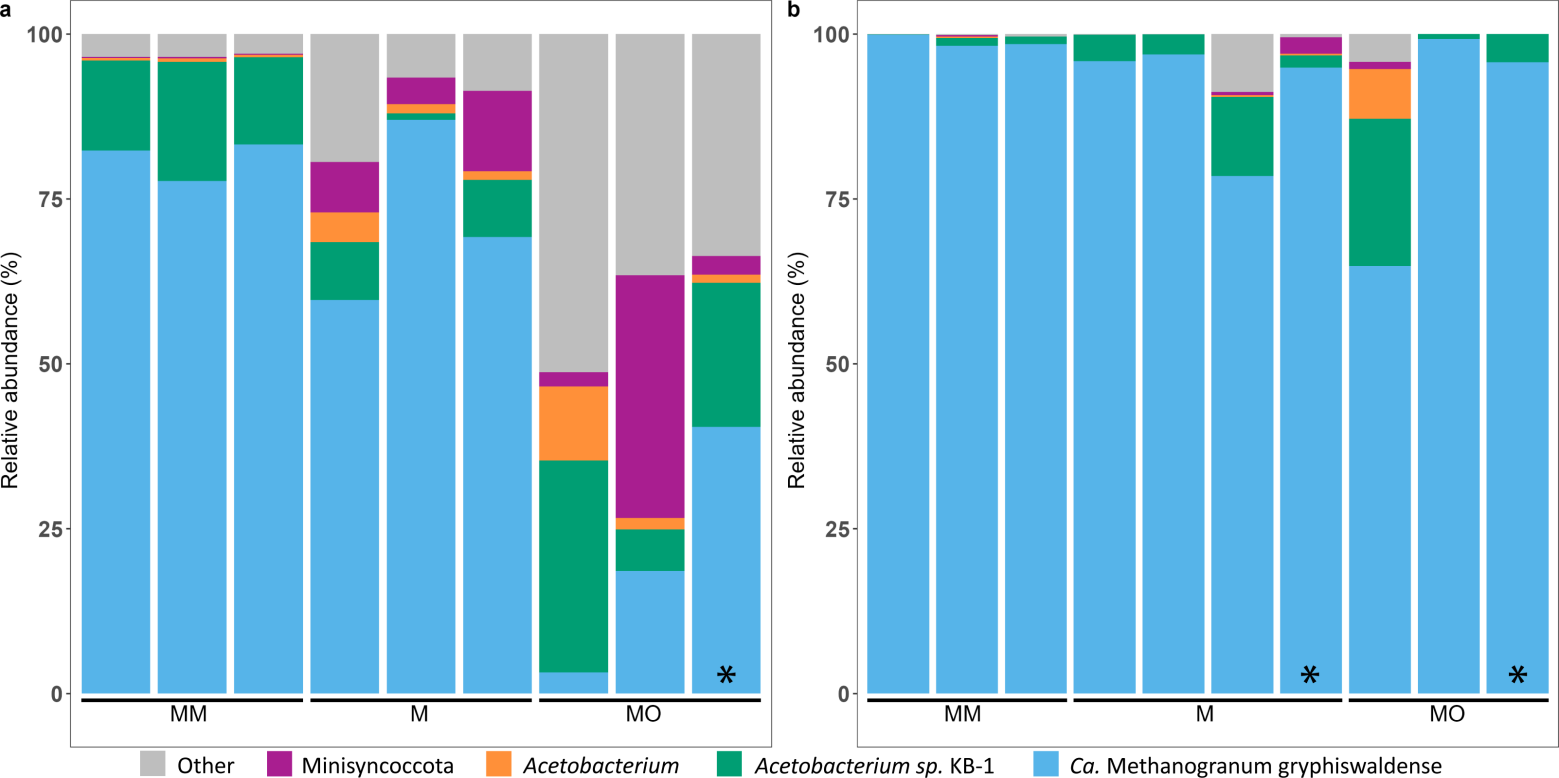
Microbial community composition. (**a**) Community composition according to mapping the quality-filtered reads to the de-replicated MAGs. (**b**) Community composition according to metatranscriptomes. Taxonomic assignment was on species level for *Ca.* Methanogranum *g*ryphiswaldense and *Acetobacterium* sp KB-1, genus level for *Acetobacterium*, and order level for Minisyncoccota while grouping low-abundant clades as “other.” * denotes samples taken from second incubation as indicated in Fig. 2.

The MM, M, and MO methane-producing cultures were targeted for metagenomic and metatranscriptomic analyses to investigate their genetic potential and mRNA transcript-based metabolic activities. They were sampled at day 160 for M and MO cultures and day 126 for cultures MM, when increasing δ^13^C values indicated methanogenesis was still ongoing. Samples from the second incubation, indicated by M* and MO*, were taken when methane production had begun to decline. The nucleic acid sequencing generated a total of 33 metagenome-assembled genomes (MAGs) from nine metagenomic samples (Table S5). Among these MAGs, 32 and 1 were classified as Bacteria and Archaea, respectively (Fig. 3, further taxonomic levels in Fig. S6).

### Enrichment culture microbial community composition

The MAG with the highest relative abundance (3.2% to 87%) was classified as the archaeon *Methanogranum* sp019262145, also known as *Candidatus* Methanogranum gryphiswaldense (ANI threshold 95%; Fig. 3). This Methanomassiliicoccales order archaeon has previously been detected in peatland soils, freshwater and marine sediments, deep-sea hypersaline anoxic basins, geothermal hot springs, and in the deep biosphere (30–33). Members of this order are described as coding for a distinctive H_2_-dependent methyl-reducing methanogenesis pathway (34). The *Acetobacterium* sp. KB-1 MAG (Acetobacterium sp003260995; ANI threshold 95%) was originally described from an anaerobic bioreactor (35) and constituted the second most abundant population in most of the enrichment cultures, ranging from 1% to 32%. The single Minisyncoccota MAG in all cultures was C*andidatus* Saccharibacteria UBA1547 (from 0.2% to 36.8%), which had its highest relative abundances in cultures supplemented with oil. Saccharibacteria have an ultra-small cell size and are obligate symbionts or parasites to other bacteria, critically relying on their hosts for essential metabolites and cellular components, including DNA and RNA precursors (Text S1).

Certain MAGs were only found in cultures supplemented with oil, such as *Yersinia intermedia* (up to 1.8%) that is a facultative anaerobe capable of fermentation in the absence of oxygen (36). Máthé et al. (37) reported that a *Y. intermedia* isolate degrades a broad spectrum of aromatic hydrocarbons, such as benzene, naphthalene, and *n*-dodecane. Other taxa that potentially utilize hydrocarbons and were solely present in the MO cultures were Acidanimobacteraceae (38), *Clostridioides mangenotii* (39), and *Aminipila,* which can play a role in biomass recycling (40), indicating that the presence of oil impacts community structures and species abundances. This was also supported by species richness being higher in MO treatments compared with treatment M and MM, with the latter being least rich, though no significant difference was measured (Fig. S7).

Metatranscriptomic analysis identified RNA transcripts from nine MAGs, indicating they were transcriptionally active at the time of sampling. These MAGs included *Sphaerochaeta*, *Proteiniclasticum*, *Acetobacterium* sp. KB-1, *Humidesulfovibrio*, *Paludibacter* sp018054805, *Acetobacterium*, *Ca*. Methanogranum gryphiswaldense, UBA1547, and *Sedimentibacter. Ca*. Methanogranum gryphiswaldense was the dominant MAG and contributed up to 99.9% of the total RNA transcripts in MM cultures (Fig. 3b). Moreover, transcripts were assigned to *Ca*. Methanogranum gryphiswaldense and *Acetobacterium* sp. KB-1 in every sample, with 0.1% to 22.3% of transcripts assigned to *Acetobacterium* sp. KB-1 (Fig. 3b). Overall, *Ca*. Methanogranum gryphiswaldense and *Acetobacterium* sp. KB-1 had the highest relative abundances of RNA transcripts in M and MM cultures, while the cultures supplemented with oil also enriched additional populations, decreasing the relative abundance of *Ca*. Methanogranum gryphiswaldense and *Acetobacterium* sp. KB-1.

### Metabolic potential and dominant functions of the methane-producing consortia

**Fig 4:**
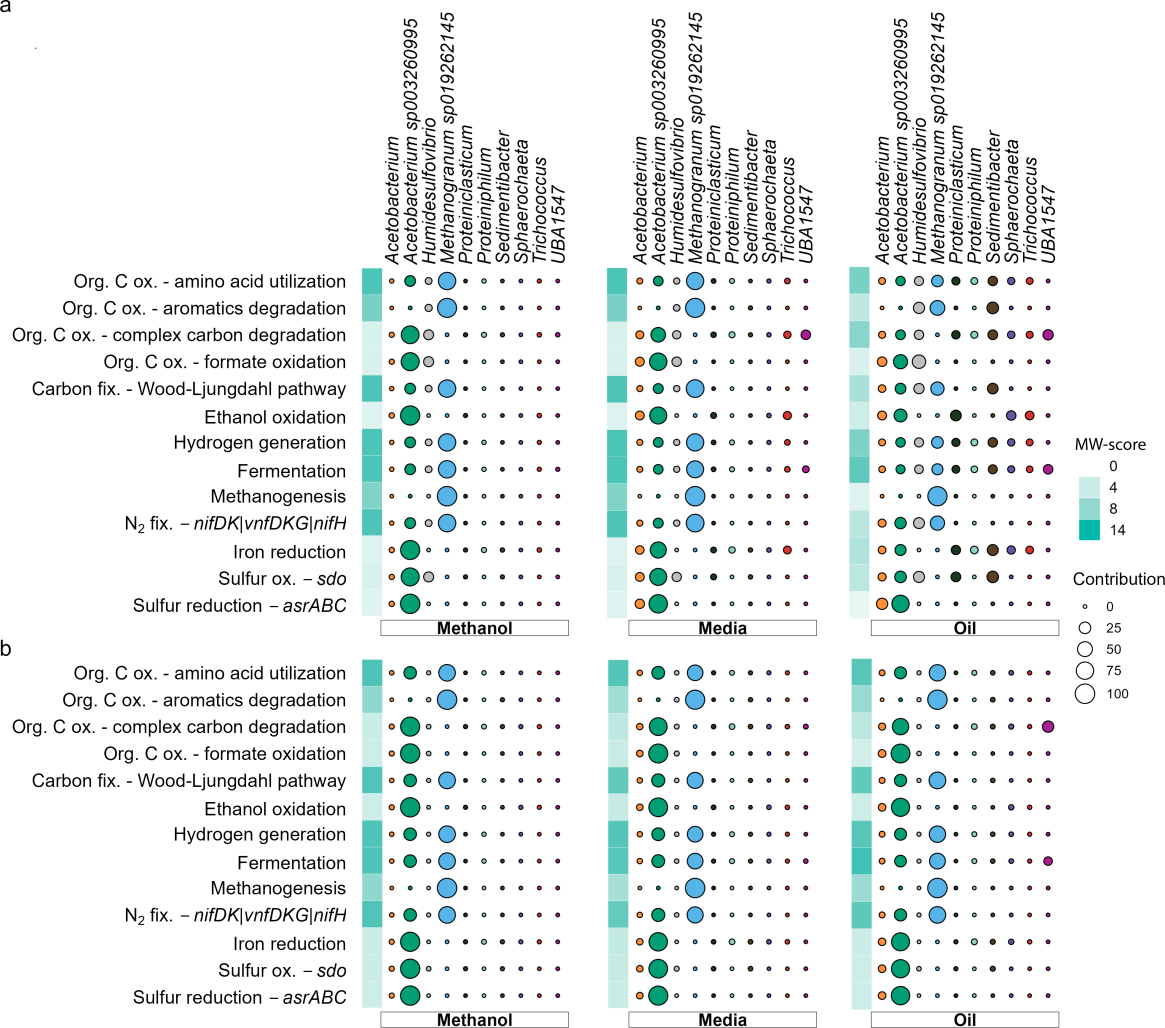
Relative contribution of taxa across selected metabolic functions. Metabolic weight of the most abundant populations, according to MAGs (**a**) and metatranscriptomes (**b**). The metabolic weight score quantifies the contribution of a specific population to a metabolic function, with MW-score per sample ranging from 0 to 14 and population contribution ranging from low (0) to high (100), with the different taxa represented by color.

The metabolic potential of the methane-producing consortia was analyzed using metabolic weight scores (MW-score) of different functions (41). The most dominant function was fermentation in all enrichment cultures, averaging a score of 12. The methanogenesis MW-score was strongly correlated with the abundance of *Ca*. Methanogranum gryphiswaldense reads in the metatranscriptomes, with the MM and M treatments showing MW-scores of 6.7 to 11.4 as compared with 0.3 to 7.4 in the MO metatranscriptomes. In the oil-amended cultures, a larger number of taxa were present performing various functions (Fig. 4), while the *Ca*. Methanogranum gryphiswaldense methanogen was present in low abundance (Fig. 3). The complex carbon compounds provided by the oil resulted in more relatively abundant taxa in the MO treatment that could grow via fermentation. Moreover, sulfate-reducing bacteria can outcompete methanogens for substrates like hydrogen and acetate at dissolved sulfate concentrations >60 µM (42). This competition likely resulted in the different functional dominance in the different treatments.

### Functional activity and specific pathways of the methanogenic consortia

The MW-score can be further analyzed by examining the relative contribution of each taxon to the overall score for each function. The most dominant functions were associated with the two most dominant and transcriptionally active MAGs, *Ca*. Methanogranum gryphiswaldense and *Acetobacterium* sp. KB-1. Specific pathways associated with *Ca*. Methanogranum gryphiswaldense were methane production, nitrogen fixation, and degradation of aromatic compounds. In the assigned RNA transcripts, the pathway for H_2_-dependent methyl-reduction was found, whereas pathways for utilization of methylamines (e.g., *mtbA*) or fermentation of acetate to methane were not found in the *Ca*. Methanogranum gryphiswaldense MAG (Fig. S8). Based on KEGG orthology (KO) identifiers (Data S4), *mtaABC* is part of the methyltransferase metabolizing methanol and transferring a methyl group to Coenzyme M, forming Methyl-CoM, which is then reduced to methane and coenzyme M-coenzyme B heterodisulfide (COM-S-S-COB) in a reaction with coenzyme B by the *mcrABG* genes. The process in which CoB-S-S-CoM is reduced back to Coenzyme M and Coenzyme B is coupled to the reduction of ferredoxin and is H_2_ dependent (EC. 1.8.98.5). Within the methylotrophic methanogenesis pathway, the distinction between methyl-dismutation (disproportion) and methyl-reduction depends on the presence of the methyl branch of the Wood-Ljungdahl pathway, which is linked to the capability to oxidize methyl-CoM to CO_2_ (43, 44). As this was not detected in the *Ca*. Methanogranum gryphiswaldense MAG, the methyl-reducing pathway was the most likely metabolic pathway to generate methane using hydrogen and methanol as electron donor and acceptor, respectively.

The *Acetobacterium* sp. KB-1-assembled genome showed similar degrees of transcriptional activity in all treatments: complex carbon degradation (average MW-score 4.3 across all treatments), formate oxidation (3.9), the Wood-Ljungdahl pathway (11), and ethanol oxidation (4.3) (Fig. 4). In addition, both the metagenomes and metatranscriptomes suggested that *Acetobacterium* sp. KB-1 utilized the carbonyl branch of the Wood-Ljungdahl pathway (*cooS*, *acsB*, *cdhE*, and *cdhD*) to assimilate CO_2_ into acetyl-CoA and subsequently, through the phosphate acetyltransferase-acetate kinase pathway (*ackA*). Ross et al. (44) described the metabolic potential of *Acetobacterium* sp. KB-1 for ethanol oxidation and 2,3-butanediol metabolism, indicating the possible pathway of ethanol as an electron donor to produce acetate and hydrogen.

In this methanogenic consortium, the most dominant and active population, *Ca*. Methanogranum gryphiswaldense, generated methane via a methyl-reduction pathway, but the source for the H_2_ supply necessary for this pathway remains elusive and might involve cryptic cycling.

### Cryptic hydrogen cycling

**Fig 5:**
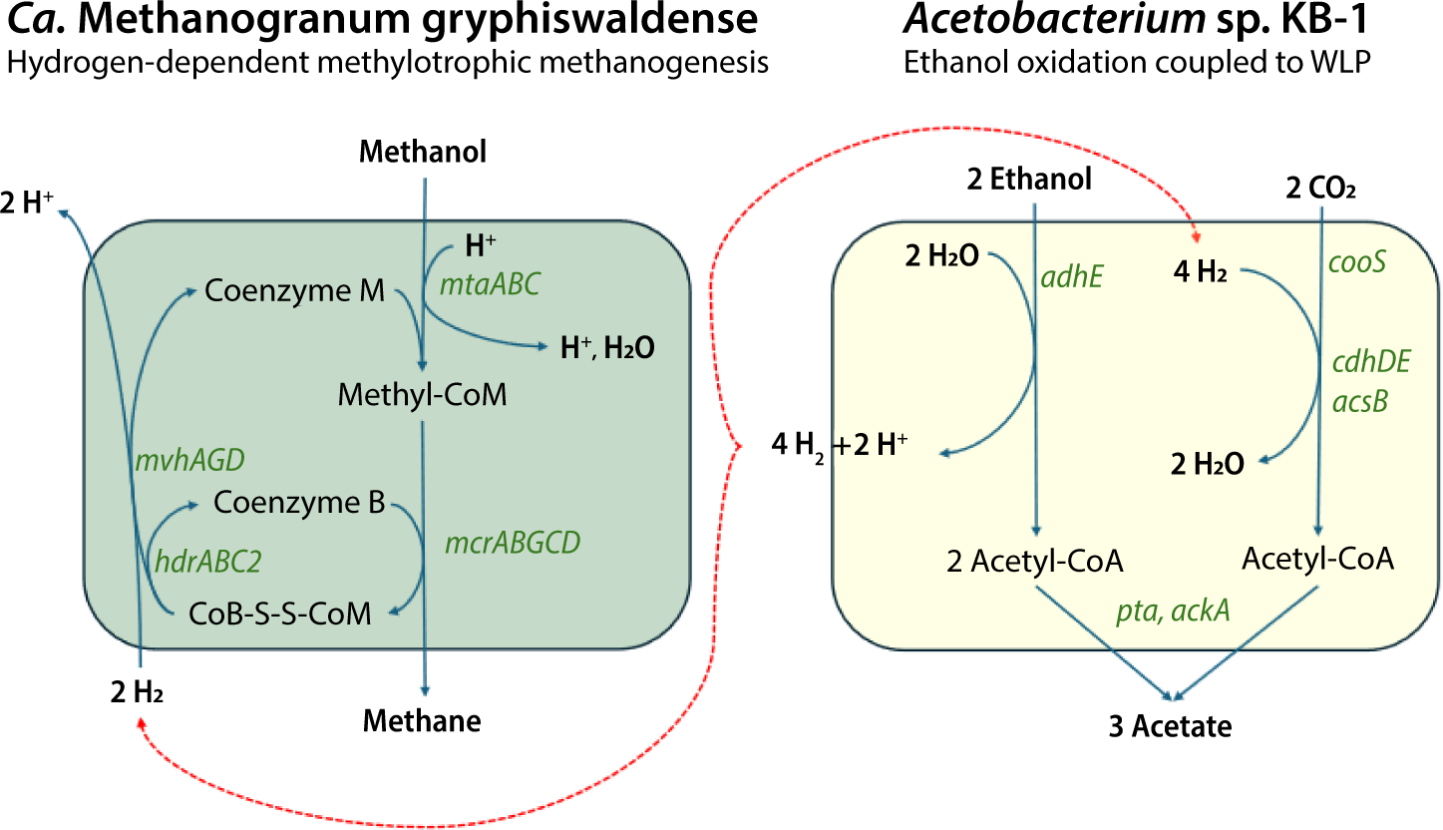
Conceptual model for interspecies hydrogen transfer between *Ca*. Methanogranum gryphiswaldense and *Acetobacterium* sp. KB-1. The hydrogen generated through ethanol oxidation is partly used for hydrogen-dependent methanogenesis and partly used for the Wood-Ljungdahl pathway (WLP), indicated by red dashed arrows. The genes (in green) were all identified in the transcripts, except for *pta* and *mvhA* that were identified in the genome.


Cryptic cycling refers to the process of element cycling often occurring in low carbon and energy environments, where electron donors will not accumulate but be consumed immediately. Therefore, potential electron donors will not appear in measurements and their cycle will be largely “invisible” (45). This work, as well as previous studies (33), provides evidence that *Ca*. Methanogranum gryphiswaldense was restricted to H_2_-dependent methylotrophic methanogenesis. Pure cultures of *Methanomassiliicoccus luminyensis*, the closest described member of the *Ca*. Methanogranum genus, can only grow when amended with both methanol and H_2_ (46). Remarkably, the enrichments in this study grew without an exogenous H_2_ supply and no H_2_ was detected in the headspace (Table S3). However, the metagenomic analysis revealed that the *Ca*. Methanogranum gryphiswaldense MAGs lacked the Wood-Ljungdahl pathway and F_420_ enzyme, limiting their metabolic route to the methyl-reduction pathway, which requires H_2_ to produce COM-S-S-COB. Therefore, the molecular hydrogen must be supplied by another microbe, possibly in a syntrophic relationship with *Ca*. Methanogranum gryphiswaldense. While fungi may be potential candidates for providing H_2_ in the deep biosphere (19), the low abundance of eukaryotes in the cultures suggested that such organisms would not be significant contributors to hydrogen production (Fig. S6). Another possible pathway of hydrogen production is through fermentation of dead biomass (necromass) by fermentative bacteria in the culture. This could generate H_2_, which could in turn be used by the methanogen. *Acetobacterium* sp. KB-1, the second most abundant taxa in the cultures, typically consumes H_2_ to produce acetyl coenzyme A (acetyl-CoA) from CO_2_ and produce acetate via the Wood-Ljungdahl pathway (47). To reduce CO_2_, acetogens can use H_2_ as an electron donor, but due to their high metabolic versatility, they can also utilize many other substrates (48). If H_2_ is scarce, or if acetogens are outcompeted for H_2_, they can use different substrates for both energy and carbon, providing a competitional advantage (48–50). The metatranscriptomes indicated that *Acetobacterium* sp. KB-1 and *Acetobacterium* sp. both transcribed genes encoding 2,3-butanediol metabolism (*acoRABCL*) and ethanol oxidation (*adhE*). Ethanol oxidation was an important function for *Acetobacterium* sp. KB-1, as well as high activity in the Wood-Ljungdahl pathway for *Acetobacterium* (*cooS;* Fig. 4; Fig. S8). Ethanol can be produced by several families in the enrichment cultures, such as *Oscillospiraceae* (51), *Sphaerochaeta* (52), and members of *Clostridiaceae* (53), in which the presence of *adhE* in the MAGs was determined. These bacteria can also play a role in the production of H_2_. Both ethanol and 2,3-butanediol can be used as electron donors by *Acetobacterium* sp. KB-1 instead of H_2_, resulting in H_2_ production that can in turn be used for CO_2_ reduction (48). However, this pathway is only energetically favorable if there is a partner organism, like *Ca*. Methanogranum gryphiswaldense, that consumes H_2_ (Fig. 5). This is because ethanol oxidation to acetate and H_2_ is an endergonic reaction (Reaction 2) and thus, unfavorable and unable to support growth of a single organism (ΔG′° +19 kJ per 2 mol ethanol [54]). A syntrophic partner organism that immediately lowers the H_2_ concentration shifts the redox potential by consuming the H_2_ product and thus overcoming the thermodynamic barrier (54) by using H_2_ and methanol for methane production (Reaction 2; ΔG′° −113 kJ mol^−1^ CH_4_ [55]) to make the potential complete reaction exergonic with an ΔG′° of −94 kJ mol^−1^ CH_4_ (Reaction 3).

(1) $$CH_{3}OH+H_{2}\to CH_{4}+H_{2}O$$

(2) $$2CH_{3}CH_{2}OH+2H_{2}O\to 2CH_{3}COO^{-}+2H^{+}+4H_{2}$$

Potential reaction:


(3) $$2{\text{CH}}_{3}{\text{CH}}_{2}\text{OH}+{\text{CH}}_{3}\text{OH}+{\text{H}}_{2}\text{O}\to 2{\text{CH}}_{3}{\text{COO}}^{-}+2{\text{H}}^{+}+{\text{CH}}_{4}+3{\text{H}}_{2}$$

Bryant et al. (56) first described this syntrophic relationship between an acetogen using ethanol as electron donor and a hydrogenotrophic methanogen that lowers the H_2_ concentration. However, this would not be restricted to ethanol oxidation as acetogens can use multiple substrates, even simultaneously (57), such as 2,3-butanediol, formate, and more complex carbon, such as glucose or fructose (Fig. 4). Besides *Acetobacterium*, several other taxa present in the cultures encode hydrogen production. Comparing the hydDB-identified hydrogenase groups (58, 59) to the transcripts, *Acetobacterium* (KB-1), *Ca*. Methanogranum, and *Sphaerochaeta* contained [NiFe] group 4f. The role of this group has not been fully determined but has been associated with oxidation of formate (or other one-carbon compounds) to H_2_ evolution and proton translocation (58). In addition, Trichococcus and Sedimentibacter contained [FeFe] group B, which is suggested to couple oxidation of ferredoxin to fermentative evolution of H_2_ (59, 60).

The presence of MAGs of these taxa in the cultures revealed that Acetobacterium was the most the most abundant hydrogen producer in the enrichment culture. *Sphaerochaeta* and *Sedimentibacter* were not present in every replicate, Tricococcus was present with an abundance of 0.05% to 4.2%, whereas the two taxa of Acetobacterium had a relative abundance of 2.4% to 43.3% summed. Given their presence and abundance in every replicate of every treatment, the metabolic versatility of *Acetobacterium* sp. KB-1 and their potential syntrophic partnership may have allowed *Ca*. Methanogranum gryphiswaldense to thrive and produce methane.

These results provide insights into potential deep biosphere methanogenic pathways. Although this enrichment was biased towards heterotrophic methanogenesis by providing organic substrates in the medium, substrates such as methanol can also be present in the deep biosphere as a product from other microbial processes. For instance, Huang et al. (61) described how subsurface-derived *Zhaonella formicivorans* converts formate into methanol if a syntrophic partner organism is present that consumes methanol, thereby providing a source of methylated compounds in the deep subsurface for methylotrophic methanogenesis. Methanol can also form as a byproduct of microbial demethylation reactions (62), from degradation of pectin (63), and as a product of methanotrophic bacteria using methane as a substrate (64). This study successfully enriched a methanogenic consortium, confirming active methanogenesis from communities enriched from groundwater in the Siljan terrestrial impact crater.

## MATERIALS AND METHODS

### Groundwater sampling

The Siljan impact crater sampling was performed in September 2021 from approximately 380-m depth in a borehole in the sedimentary ring around the crater (Fig. 1) (20). Briefly, the borehole section volume was pumped and allowed to refill three times prior to sampling to avoid stagnant water, and then groundwater was captured in acid-washed, nitrogen-flushed, sterile glass bottles and allowed to overflow with at least three volumes to remove any free oxygen such that the cells were maintained under anoxic conditions. In addition, oil was sampled from a nearby borehole using a sterile 50-mL Falcon tube (20). Samples were cooled during transport to the laboratory before being transferred to an anaerobic chamber for preparation of the enrichment cultures.

### Microbial enrichment cultures

DSMZ medium 120 for *Methanosarcina* for the enrichment cultures (Table S6) was prepared by dissolving all the ingredients except bicarbonate, vitamins (sterile filtered 0.1 µm pore size), methanol, cysteine, and sulfide in MilliQ ultrapure water, the solution was flushed with 80% N_2_ and 20% CO_2_ gas mixture for 30–45 min, and then bicarbonate was added, and the medium autoclaved at 121°C for 20 min. In addition, methanol and the reducing agents were prepared under 100% N_2_ gas atmosphere and autoclaved separately. The solutions were transferred to an anaerobic chamber (MBraun Labstar, H_2_O and O_2_ purity below 1 ppm), and 50-mL aliquots were dispensed into 100-mL sterile serum bottles. The bottles were closed with a 20-mm thick butyl stopper and sealed with aluminum crimp caps. Using hypodermic syringes, 10 mL of groundwater (as inoculum) was added to each bottle containing the different substrates as described in Table S1. The solution pH was adjusted to 6.5–7 using NaHCO_3_. In addition, further enrichment cultures were inoculated with different electron donors (methanol, acetate, yeast extract, and casein) and inoculated with the M cultures from the first experiment. Controls consisted of groundwater or media only. All cultures were kept in a temperature-controlled room at 14°C, which closely matches the *in situ* temperature of the groundwater.

### Gas sampling and analysis

The culture headspace methane concentration was measured by taking a gas sample with a syringe and injecting it into a ThermoFisher Trace 1310 gas chromatograph (GC) equipped with a flame ionization detector (FID) with a PoraPLOT-Q 25 m × 0.32 mm capillary column and operations conditions of 80°C (oven) and 250°C. Helium was used as a carrier gas at a constant rate of 5 mL min^−1^ and reference gases contained 10 ppm, 100 ppm, 1,000 ppm, and 1% and 10% methane diluted in helium. Stable isotopic analyses of methane were performed in samples when methane concentrations reached >1,000 ppm in a ThermoFisher Delta V Plus Isotopic Ratio Mass Spectrometer coupled to a ThermoFisher GC-Isolink and a Trace GC 1310 with a PoraPLOT-Q 25 m × 0.32 mm capillary column (oven temperature 30°C).

Methane concentration data were analyzed using linear mixed-effects models to evaluate treatment effects over incubation time. All analyses were conducted in R (version 4.4.2). The MO treatment from first incubation was split into “high” and “low” based on clustering of average methane concentrations. Models included treatment, time, and their interaction as fixed effects, with a random intercept for each replicate to account for repeated measurements. Models were fitted using the lme4 package, and the significance of fixed effects was assessed with Type III ANOVA. Pairwise comparisons between treatments were performed with Tukey’s adjustment (*P* < 0.005).

Headspace gas samples were analyzed by Applied Petroleum Technology, Oslo, for full chemical and isotope compositions (Tables S2 and S3). Hydrocarbons were identified and quantified with a GC equipped with FID and H_2_, CO_2_, N_2_, and O_2_/Ar with a thermal conductivity detector. The GC for hydrocarbon analysis was an Agilent 7890 A instrument. The chromatographic column was an HP PONA (50-m length, 0.2-mm internal diameter, 0.5-µm film thickness). The temperature program of the GC-oven started at 30°C (held for 10 min), followed by heating to 60°C (held for 10 min) with 2 °C min^−1^, heating to 130°C with 2 °C min^−1^, and heating to 320°C (held for 25 min) with 4 °C min^−1^. 2,2,4-Tri-methyl-pentane was used as an internal standard. The stable carbon isotopic composition of the hydrocarbon components in the gas was determined by a GC coupled to a combustion and isotope ratio mass spectrometer, with a reproducibility of δ^13^C values better than 1 ‰ V-PDB (two sigma). The methane hydrogen isotope composition was determined with a reproducibility of δD values better than 10 ‰ V-SMOW (two sigma).

Free gas methane-clumped isotopologues were measured at the Massachusetts Institute of Technology by extracting and purifying the methane from gas samples following the preparative GC method described by Wang et al. (65). The relative abundances of methane isotopologues ^12^CH_4_, ^13^CH_4_, ^12^CH_3_D, and ^13^CH_3_D were measured using a tunable infrared laser direct absorption spectroscopy (TILDAS) as previously described (66). TILDAS values of δ^13^C and δD of methane were calibrated via measurements of natural gas standards NGS-1 and NGS-3 (65). The abundance of ^13^CH_3_D isotopolog was reported as ∆^13^CH_3_D, a metric representing the deviation of the abundance of ^13^CH_3_D from a stochastic distribution of isotopes among isotopologs ^12^CH_4_, ^13^CH_4_, ^12^CH_3_D, and ^13^CH_3_D:


$$\Delta^{13}CH_{3}D=\frac{{[}^{13}CH_{3}D]\;{[}^{12}CH_{4}]}{{[}^{13}CH_{4}]\;{[}^{12}CH_{3}D]}-1$$

The stochastic isotopolog ratios (i.e., Δ^13^CH_3_D = 0) were determined by thermally equilibrating methane at 250°C using Pt catalyst (66) and the theoretical fractionation factors of (67).

### DNA/RNA extraction

DNA and RNA were sampled on days 126 and 160 from 1.8 mL of MM and M plus MO cultures, respectively, and extracted using Qiagen’s AllPrep DNA/RNA/Protein Mini Kit, following the manufacturer’s protocol. The libraries for metagenomic sequencing were prepared using the Tecan MagicPrep, adjusting the number of cycles (four or seven) to the amount of template DNA added (80 or 300 ng). DNA concentration in the amplified libraries was quantified with a Qubit 3 fluorometer (Life Technologies). The libraries were pooled to an equimolar concentration of 10 ng µL^−1^, and the fragment length of the pooled libraries was checked using agarose gel electrophoresis. The extracted RNA was treated with DNase followed by ribosomal RNA depletion using Illumina’s RiboZero Plus kit. The libraries were chemically fragmented and cDNA libraries generated using reverse transcriptase. Prior to sequencing, the fragment size distribution of the cDNA libraries was evaluated with a Fragment Analyzer 5300 (Agilent), and the concentration was determined using qPCR while targeting the ligated Illumina adapters.

Both library types (DNA and cDNA) were sequenced at the Science for Life Laboratory (SNP&SEQ platform) in separate lanes on an Illumina NovaSeq 6000 equipped with a SP-300 flow cell, producing 2 × 150 bp paired-end reads.

### Bioinformatics

Raw sequences from the metagenomes (*n* = 9) were processed using the curated nf-core/mag pipeline (version 2.2.1 [68]) on default settings, unless otherwise specified (69). Briefly, quality filtering and adapter trimming were done with fastp (v0.23.2), and the reads were assembled into contigs using MEGAHIT (v1.2.9), followed by evaluation of the contigs with Quast (v5.0.2). Open reading frames were predicted with Prodigal (v2.6.3). Prokka (v1.14.6) and eggNOG-mapper (v2.1.9) were used for functional annotation. The contigs were binned with MetaBAT2 (v2.15), and the generated bins were refined with DAS Tool (v1.1.4), followed by evaluation of the refined bins with CheckM (v1.1.3). The bins were de-replicated with dRep (v3.4.2) while maintaining a maximum contamination of 5% and a minimum completeness of 70%. GTDB-Tk (v2.1.1) was used for taxonomic assignment, followed by quantifying read coverage (according to transcripts per million) by mapping the quality-filtered reads to the de-replicated bins with CoverM (v0.6.1).

The metatranscriptomes (*n* = 9) were mapped to the reconstructed bins (metagenome-assembled genomes) using the nf-core/magmap pipeline (v1.0dev). Briefly, the raw reads were processed by quality filtering and adapter removal with Trim Galore (v0.6.7). Ribosomal RNA was removed with BBduk (v39.01) combined with the SILVA rRNA database (v138.1), followed by aligning of the quality-filtered reads with the reconstructed bins using BBmap index (v39.01). Read coverage (in tpm) and mapping rate were obtained using FeatureCounts (2.0.1), followed by functional annotation of transcripts using Prokka (v1.14.6) and eggNOG-mapper (v2.1.12).

To evaluate both the metabolic potential and activity of the recovered MAGs across different samples, METABOLIC-C v4.0 (41) was run in “community” mode using quality-trimmed metagenomic and metatranscriptomic data sets. The tool calculates the Metabolic Weight (MW) score by summing the gene coverage for each function across genomes and normalizing it to the total functional coverage across all functions in the data set. Higher MW scores indicate more widely shared and abundant functions, reflecting dominant metabolic activities and microbial contributions (41).

The presence of hydrogenase encoding genes was evaluated using HydDB (58, 59) (https://github.com/GreeningLab/HydDB). The steps under “Simple hydrogenase classification with DIAMOND BLASTP” were followed, except Blastx was run instead of Blastp.

Alpha diversity was quantified separately for treatment M, MO, and MM. Estimated richness was calculated using the Chao1 index to account for unobserved taxa and group comparisons were performed using the Kruskal-Wallis rank-sum test.

## Data Availability

Metagenomic and metatranscriptomic sequences are available under ENA project PRJEB85571. The R Markdown that was used to generate the figures is available at https://doi.org/10.5281/zenodo.15051407.

## SUPPLEMENTAL MATERIAL

Data S1 mbio.03017-25-s0001.xlsx Methane concentration and isotope data from first incubation.

Data S2 mbio.03017-25-s0002.xlsx Methane concentration and isotope data from second incubation.

Data S3 mbio.03017-25-s0003.xlsx Carbon dioxide concentration data from second incubation.

Data S4 mbio.03017-25-s0004.xlsx KO identifiers used for analyzing the metabolic pathways.

Supplemental Material mbio.03017-25-s0005.docx Text S1, Tables S1 to S6, and Fig. S1 to S8.

ASM does not own the copyrights to Supplemental Material that may be linked to, or accessed through, an article. The authors have granted ASM a non-exclusive, world-wide license to publish the Supplemental Material files. Please contact the corresponding author directly for reuse.
