# Supplementary material for: Active methylotrophic methanogenesis by a microbial consortium enriched from a terrestrial meteorite impact crater
Source: mBio. 2025 Nov 25;17(1):e03017-25. doi: 10.1128/mbio.03017-25 (PMC12802149; doi:10.1128/mbio.03017-25)
Supplement: Supplemental Material — Text S1, Tables S1 to S6, and Fig. S1 to S8. [file mbio.03017-25-s0005.docx]

**Active methylotrophic methanogenesis by a microbial consortium enriched
from a terrestrial meteorite impact crater**

Femke van Dam, George Westmeijer, Maryam Rezaei Somee, Marcelo Ketzer,
Riikka Kietäväinen, Shuhei Ono, Stefan Bertilsson,
Jennifer C. McIntosh, Mark Dopson, Henrik Drake

**Supplementary Materials**

**Supplementary Text 1. Saccharibacteria**

Saccharibacteria can conserve energy by fermentation [1]. In addition, Saccharibacteria can dominate enrichment cultures amended with hydrocarbons and suggest they act as scavengers rather than being directly involved in hydrocarbon degradation [2].

The C*andidatus* Saccharibacteria bacterium UBA154 was active in acetate oxidation in all samples, particularly in the treatment supplemented with oil (50 to 90% based on metatranscriptome; Fig. 4). However, cultures with treatment M, which were amended with acetate, exhibited much lower relative abundance, possibly due to competition for acetate with *Humidesulfovibrio* and *Proteiniclastricum* (Fig. 4). In MO cultures, *Saccharibacterium* was also active in degrading complex carbon, although Figueroa-Gonzalez et al. [2] suggested that *Saccharibacteria* themselves are not directly involved in the degradation of the hydrocarbons. The presence of *Saccharibacteria* in oil-polluted anaerobic environments has been often reported [3–5], since Candidate Phyla Radiation (CPR) are mostly fermentative bacteria that can survive under oil-containing environments and utilize the products of other taxa in the consortium that degrade hydrocarbons and provide smaller simple carbon compounds [2].

**Supplementary Table 1.** **Treatments and electron donors.** Conditions used for the enrichment cultures (all *n*=4) to select for methanogenic communities.

| **Treatment** | **Medium**  **DSMZ 120** | **Electron  donor** | **Groundwater inoculum** | **Enrichment^1^** |
| --- | --- | --- | --- | --- |
| Media (M)^2^ | + |  | + | 1 & 2 |
| Media + oil (MO) | + | 10 mL oil | + | 1 & 2 |
| Media^3^ + acetate | + | 30.5 mM | + | 2 |
| Media + methanol (MM) | + | 250 mM | + | 2 |
| Media + yeast extract | + | 2 mg mL^-1^ | + | 2 |
| Media + casein | + | 2 mg mL^-1^ | + | 2 |
| Groundwater control | - | - | + | 1 |
| Medium control | - | - | - | 1 & 2 |
| Oil control | - | 10 mL | - | 1 |

^1^Enrichment 1 refers to the initial cultures started from the Siljan borehole water and 2 refers to the sub-culture for further enrichment of the methane generating cultures.

^2^Acetate, methanol, yeast extract and casein are present in the Media M.

^3^Media M without methanol, yeast extract and casein.

**Supplementary Table 2.** **Headspace gas carbon isotope composition.** Headspace samples of unfiltered water (*n*=1), M culture (*n*=1), and MO culture (*n*=1) were taken day 134 of the incubation. No higher hydrocarbons were detected in M sample. Below detection limit is denoted with BD.

| **Sample information** | **C1 δ¹³C** (‰) | **C2 δ¹³C** (‰) | **C3 δ¹³C** (‰) | **i-C4 δ¹³C** (‰) | **n-C4 δ¹³C** (‰) | **CO₂ δ¹³C** (‰) |
| --- | --- | --- | --- | --- | --- | --- |
| Unfiltered groundwater | -62.6 | -29.5 | -23.9 | -29.8 | -28.6 | 6.6 |
| M culture | -69.6 | BD | BD | BD | BD | 25.3 |
| MO culture | -71.6 | -35.1 | -28.7 | -28.9 | -27.7 | 45.8 |

**Supplementary Table 3.** **Headspace gas composition data.** Samples taken from the headspace of unfiltered groundwater (*n*=1), M culture (*n*=1), and MO culture (*n*=1) at day 134 of the incubation. THCG= Total Hydrocarbon Gas.

|  | **Unfiltered groundwater** | **M culture** | **MO culture** |
| --- | --- | --- | --- |
| **C1 (%THCG)** | 18.8 | 42.4 | 6.93 |
| **C2 (%THCG)** | 0.24 | 0.0075 | 0.16 |
| **C2= (%THCG)** | 0 | 0 | 0 |
| **C3 (%THCG)** | 0.068 | 0.0044 | 3.66 |
| **C3= (%THCG)** | 0 | 0 | 0 |
| **iC4 (%THCG)** | 0.14 | 0.01 | 6.9 |
| **nC4 (%THCG)** | 0.25 | 0.017 | 9 |
| **C4= (%THCG)** | 0 | 0 | 0 |
| **neoC5 (%THCG)** | 0 | 0 | 0.13 |
| **iC5 (%THCG)** | 0.2 | 0.015 | 4.17 |
| **nC5 (%THCG)** | 0.098 | 0.0067 | 1.5 |
| **C5= (%THCG)** | 0 | 0 | 0 |
| **C6+ (%THCG)** | 1.45 | 0.056 | 1.25 |
| **CO₂ (%THCG)** | 78.7 | 57.5 | 66.3 |
| **ppm THCG** | 16021 | 41827 | 33170 |
| **H₂ (%Total)** | 0 | 0 | 0 |
| **He (%Total)** | 0 | 0 | 0 |
| **N₂ (%Total)** | 98.3 | 95.7 | 96.5 |
| **O₂+Ar (%Total)** | 0.01 | 0 | 0.11 |
| **CO (%Total)** | 0 | 0 | 0 |
| **ppm Total** | 974693 | 983500 | 983080 |
| **C1-nC4 (%THCG)** | 19.5 | 42.4 | 26.7 |
| **C2-nC4 (%THCG)** | 0.7 | 0.039 | 19.7 |
| **C5+ (%THCG)** | 1.75 | 0.077 | 7.05 |
| **Wetness** | 3.59 | 0.092 | 74 |
| **iC4/nC4** | 0.58 | 0.63 | 0.77 |

**Supplementary Table 4.** **Stable isotope ratios and clumped isotope data**. Samples of the headspace of M culture (*n*=1), and MO culture (*n*=1) taken at day 134 of the incubation and analyzed.

| **Sample** | **δ^13^C** (‰) | **std** | **δD** (‰) | **std** | **^13^CH_3_D** | **std** |
| --- | --- | --- | --- | --- | --- | --- |
| **M culture** | -61.95 | 0.23 | -292.27 | 0.26 | -4.14 | 0.28 |
| **MO culture** | -78.77 | 0.25 | -303.67 | 0.25 | -5.40 | 0.31 |

**Supplementary Table 5.** **Details of the metagenomic and metatranscriptomic sequencing generated in the study.**

| Metagenome | Million raw paired-end reads | Million QC reads | # QC de-rep MAGs | Metatranscriptome | Million raw paired-end reads | Million QC reads | QC reads mapped to MAGs (%) |
| --- | --- | --- | --- | --- | --- | --- | --- |
| VK-3516-media-gw-a_S11 | 13.2 | 13.0 | 22 | VK-3515-media-gw-a_S1 | 42.50 | 42.4 | 81.3 |
| VK-3516-media-gw-b_S12 | 7.2 | 7.0 | 22 | VK-3515-media-gw-b_S2 | 58.70 | 58.7 | 85.9 |
| VK-3516-media-gw-d_S13 | 11.8 | 11.6 | 24 | VK-3515-media-gw-d_S3 | 45.20 | 45.0 | 83.3 |
| VK-3516-media-gw-oil-b_S38 | 12.8 | 12.6 | 29 | VK-3515-media-gw-oil-b_S4 | 45.30 | 45.3 | 28.4 |
| VK-3516-media-gw-oil-c_S37 | 11.8 | 11.6 | 30 | VK-3515-media-gw-oil-c_S5 | 92.30 | 92.3 | 75.8 |
| VK-3516-media-oil-b_S28 | 10.8 | 10.6 | 20 | VK-3515-media-oil-b_S9 | 62.00 | 62.0 | 48.6 |
| VK-3516-methanol-a_S14 | 8.5 | 8.4 | 19 | VK-3515-methanol-a_S6 | 51.10 | 50.8 | 80.1 |
| VK-3516-methanol-c_S15 | 8.2 | 8.1 | 18 | VK-3515-methanol-c_S7 | 49.50 | 49.0 | 79.8 |
| VK-3516-methanol-d_S36 | 12.8 | 12.6 | 19 | VK-3515-methanol-d_S8 | 35.50 | 35.4 | 78.3 |

**Supplementary Table 6.** **Constituents of DSMZ medium 120.** Designed for the *Methanosarcina* genus and used for the initial enrichment cultures and modified for subsequent sub-cultures. Carbon sources are in bold, units are per Liter.

**DSMZ medium 120**

| **Constituent** | **Amount** |
| --- | --- |
| K_2_HPO_4_ | 0.35 g |
| KH_2_PO_4_ | 0.23 g |
| NH_4_Cl | 0.5 g |
| MgSO_4_ × 7 H_2_O | 0.5 g |
| CaCl_2_ × 2 H_2_O | 0.25 g |
| NaCl | 2.25 g |
| FeSO_4_ × 7 H_2_O | 2.00 mL |
| Trace element solution SL-10 | 1.00 mL |
| ***Yeast extract*** | 2.00 g |
| ***Casitone*** | 2.00 g |
| ***Na-acetate*** | 2.50 g |
| Sodium resazurin | 0.5 mL |
| NaHCO_3_ | 2.00 g |
| ***Methanol (50% v/v)*** | 20 mL |
| Wolin's vitamin solution (10-fold stock) | 1.00 mL |
| L-Cysteine HCl × H_2_O | 0.3 g |
| Na_2_S × 9 H_2_O | 0.3 g |
| Distilled water | To 1000 mL |

**Wolin's vitamin solution (10-fold stock)**

| **Constituent** | **Amount** |
| --- | --- |
| Biotin | 20.00 mg |
| Folic acid | 20.00 mg |
| Pyridoxine hydrochloride | 100.00 mg |
| Thiamine HCl | 50.00 mg |
| Riboflavin | 50.00 mg |
| Nicotinic acid | 50.00 mg |
| Calcium D-(+)-pantothenate | 50.00 mg |
| Vitamin B12 | 1.00 mg |
| p-Aminobenzoic acid | 50.00 mg |
| (DL)-alpha-Lipoic acid | 50.00 mg |
| Distilled water | 1000.00 ml |

**Supplementary Figure 1**. **Methane production of first incubation treatments compared to controls**. Methane concentrations across different treatments of the first incubation, separating the MO treatment in to ‘high’ and ‘low’ producing replicates. Linear mixed-effect modeling was used to reveal significant treatment x time interactions, and pairwise comparisons to controls, with asterisks indicating Tukey-adjusted *p* < 0.005.


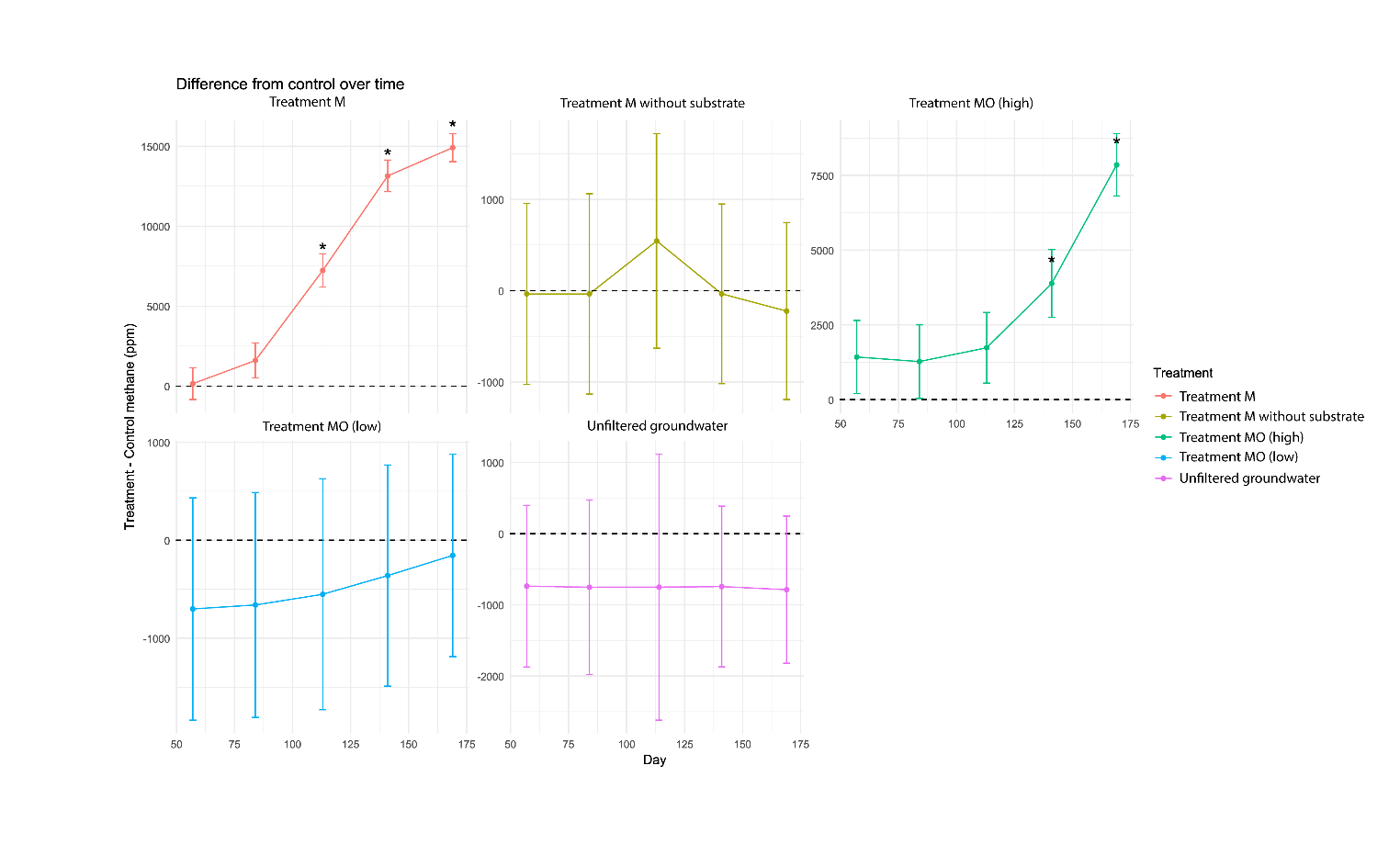


**Supplementary Figure 2**. **Methane production of second incubation treatments compared to controls**. Methane concentrations across different treatments of the second incubation. Linear mixed-effect modeling was used to reveal significant treatment x time interactions, and pairwise comparisons to controls, with asterisks indicating Tukey-adjusted *p* < 0.005.


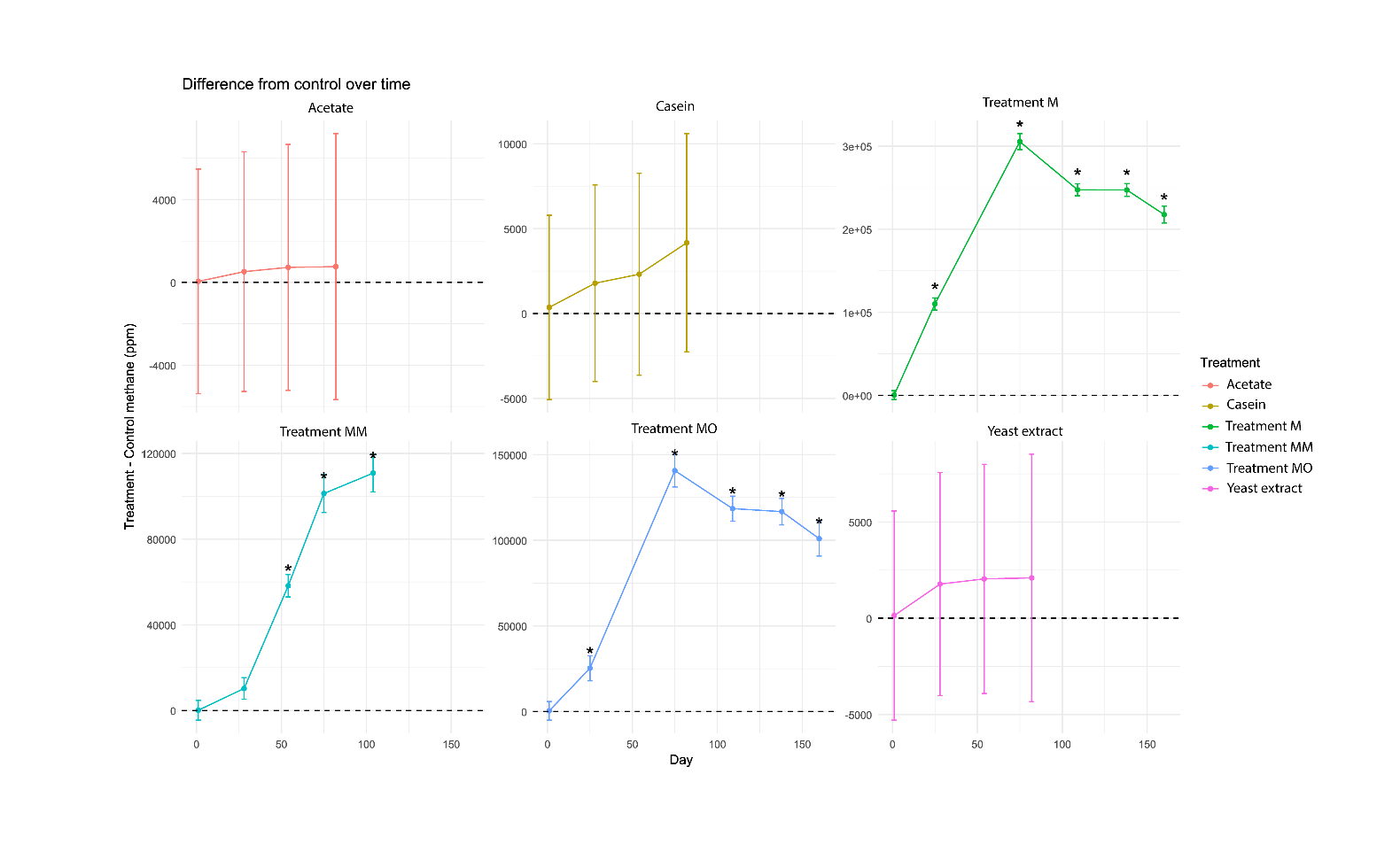


**Supplementary Figure 3**. **δ^13^C_CH4_ vs CH_4_ concentrations for Treatment MM.** Carbon isotope values increased with methane concentration. The different replicates (a-d) from the MM treatment (methanol as sole electron donor) were used to estimate the methanol to methane fractionation (ε).

**
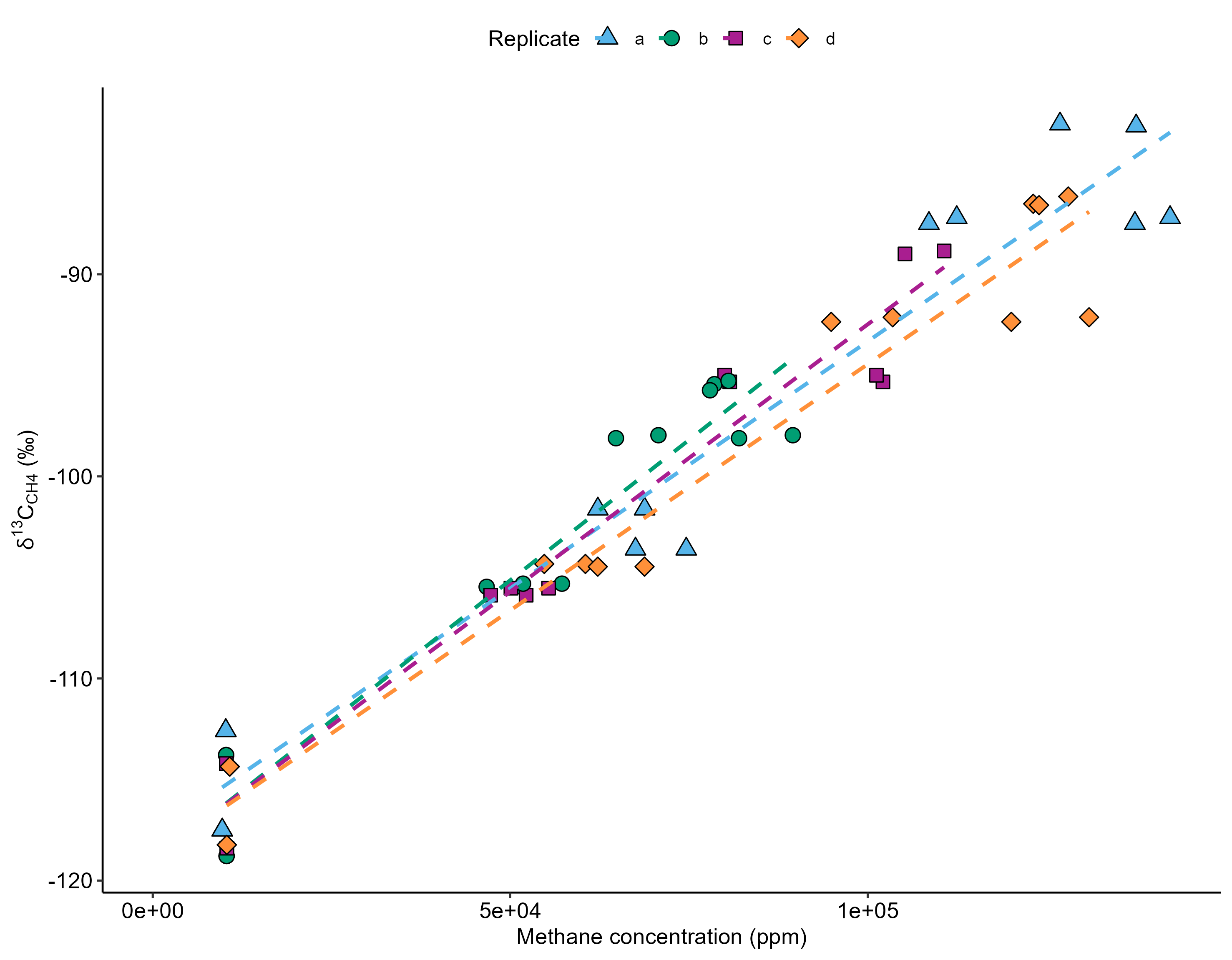
**

**Supplementary Figure 4**. **δ^13^C_CH4_ vs δD_CH4_ time-series data from the second incubation experiment**. Each treatment had *n*=4, per sub-sampling.

**
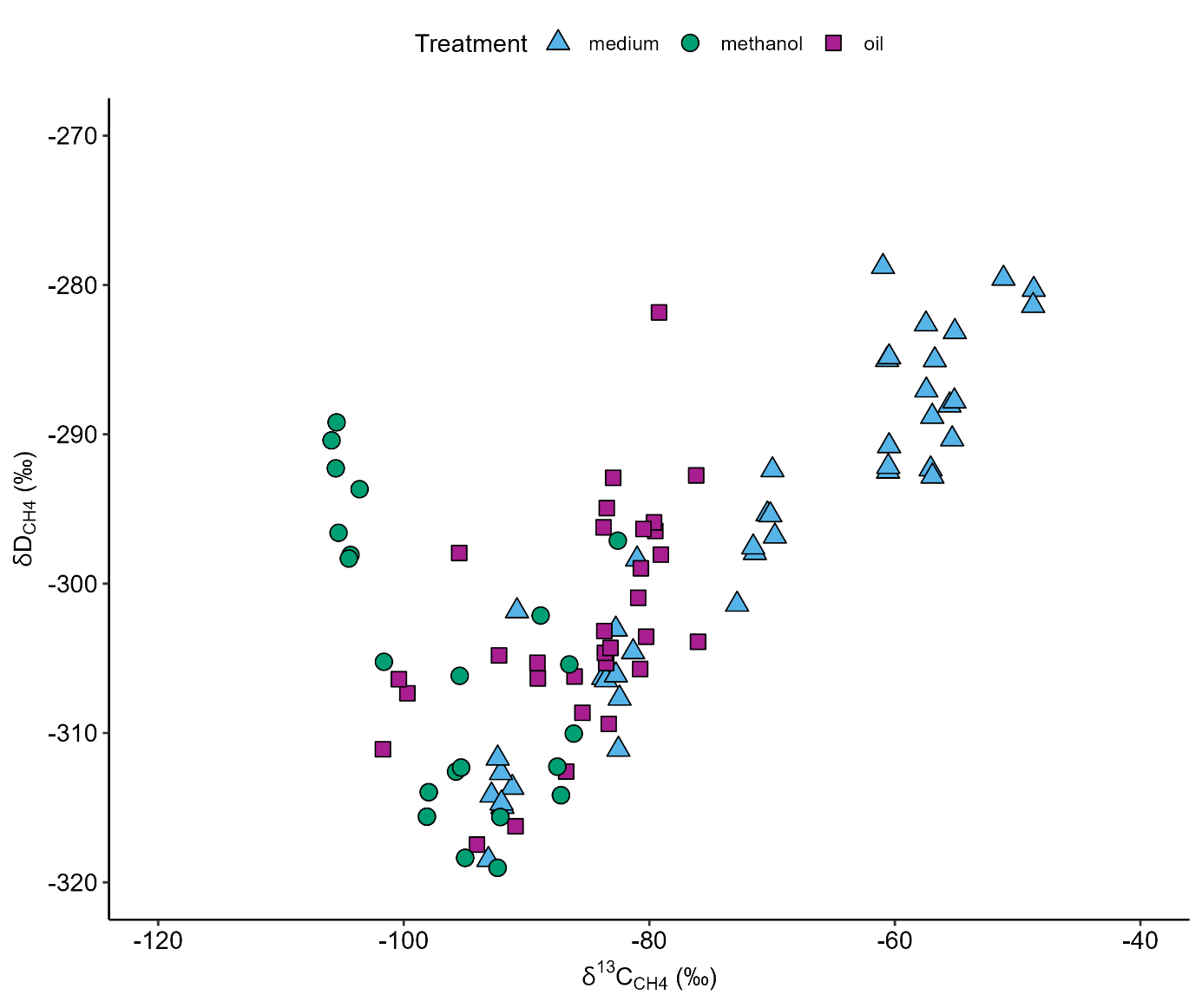
**

**Supplementary Figure 5**. **Clumped isotopes of CH_4_**. Clumped isotope data from headspace gas M and MO cultures compared to data from enrichment cultures[6–8] and natural gases[9]. Natural gas data points and fields were based on Kim et al. [9]and references therein.


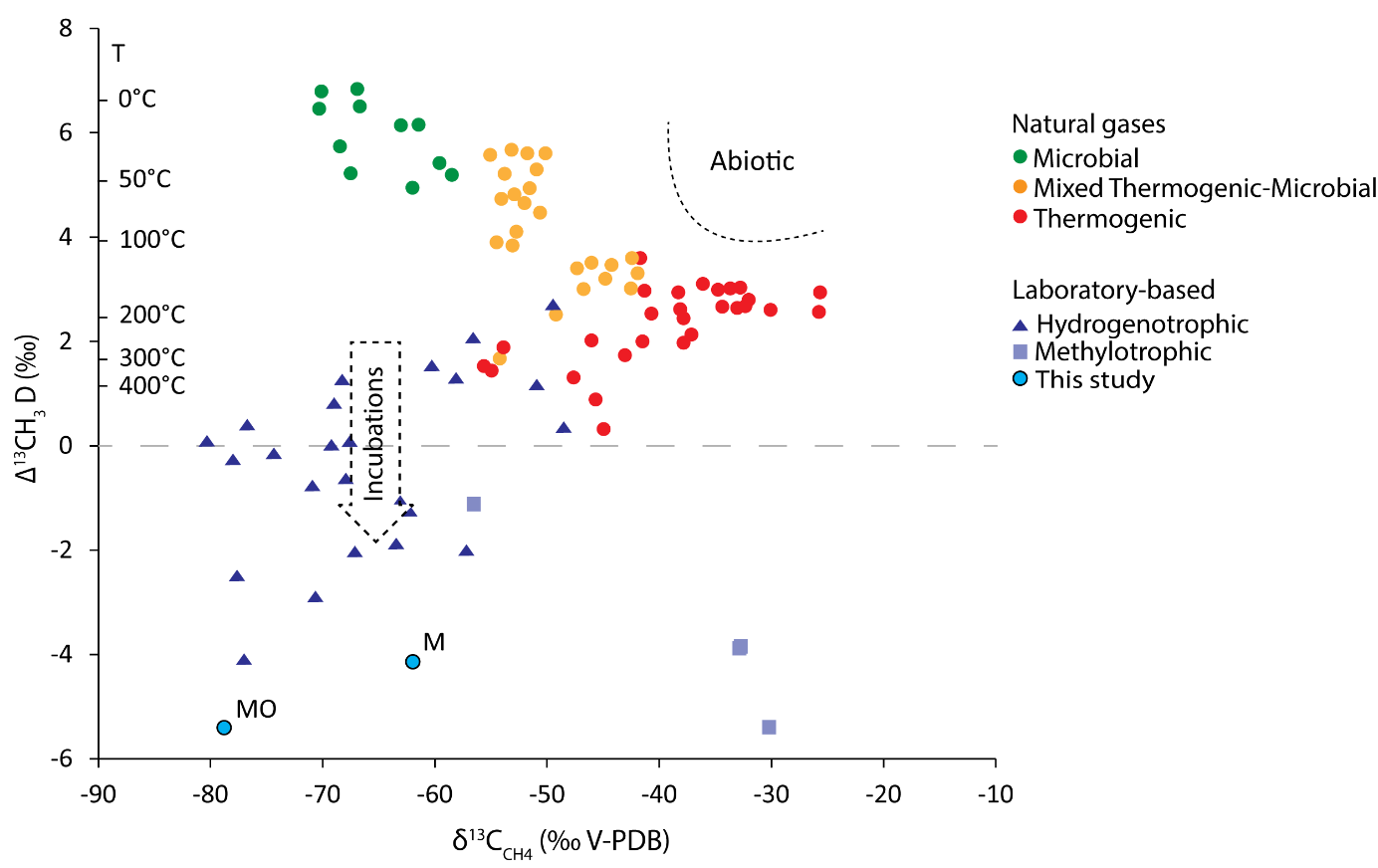


**Supplementary Figure 6**. **Microbial abundance on various taxonomic levels.** Phylum level based on a) metagenomes and b) metatranscriptomes. Family level based on c) metagenomes and d) metatranscriptomes. Genus level based on e) metagenomes and f) metatranscriptomes. The low abundance of eukaryotes precluded determination of the eukaryotic species abundance. However, a total number of 11 different species was identified: *Tritrichomonas* (Parabasalia), *Ecdysozoa nematoda, Chordata craniata, Ecdysozoa panarthropoda* (Metazoa), *Euphyllophphyta spermatophyta* (Streptophyta), *Pelogaomonas calceolata, Chattonellaceae heterosigma, Tetractinellida astrophorina* (Stramenopiles), and *Pezizomycotina leotiomyceta, Mucoromycetes Mucorales, Pezizomycotina pezizomycetes* (Fungi).

**
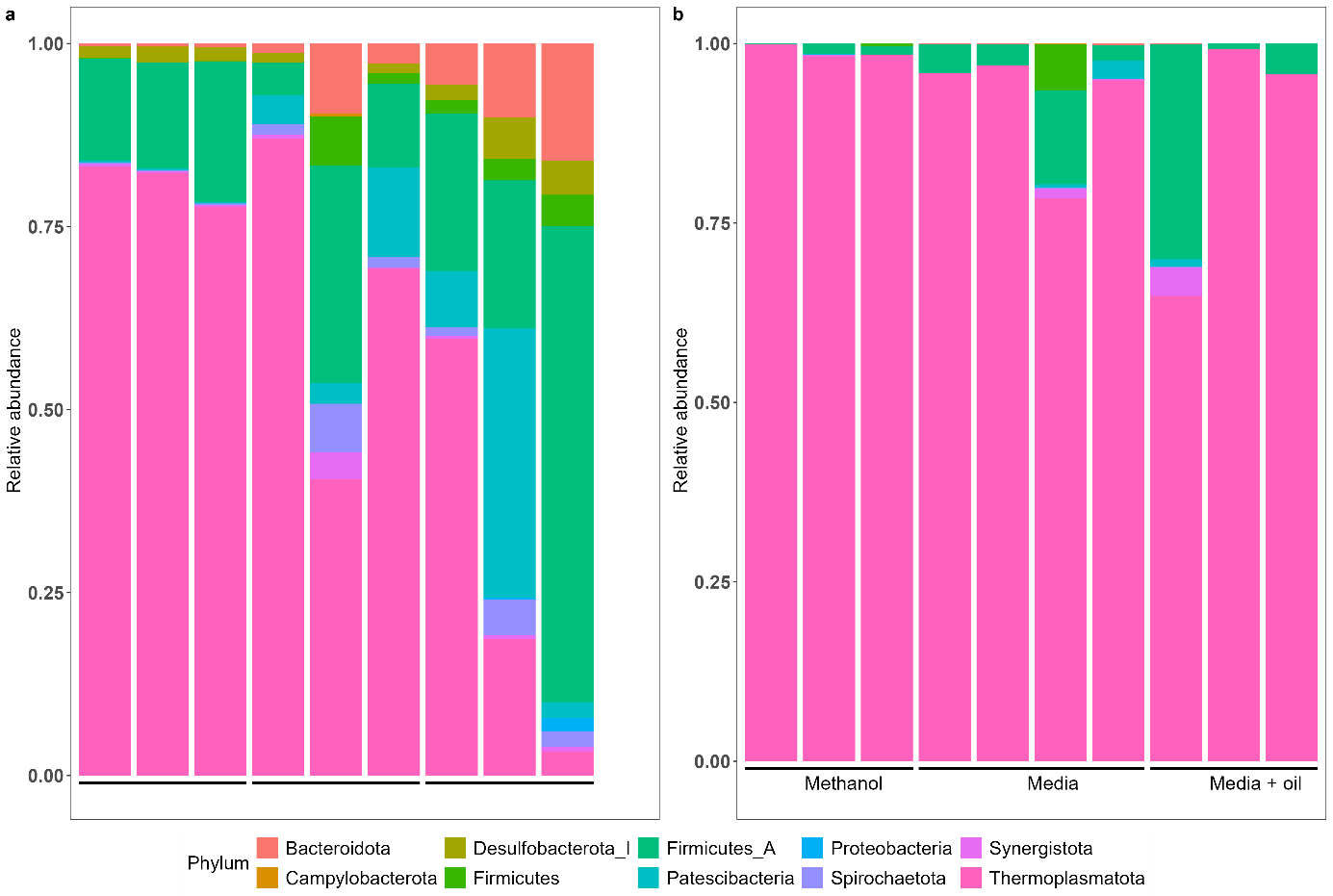
**

**
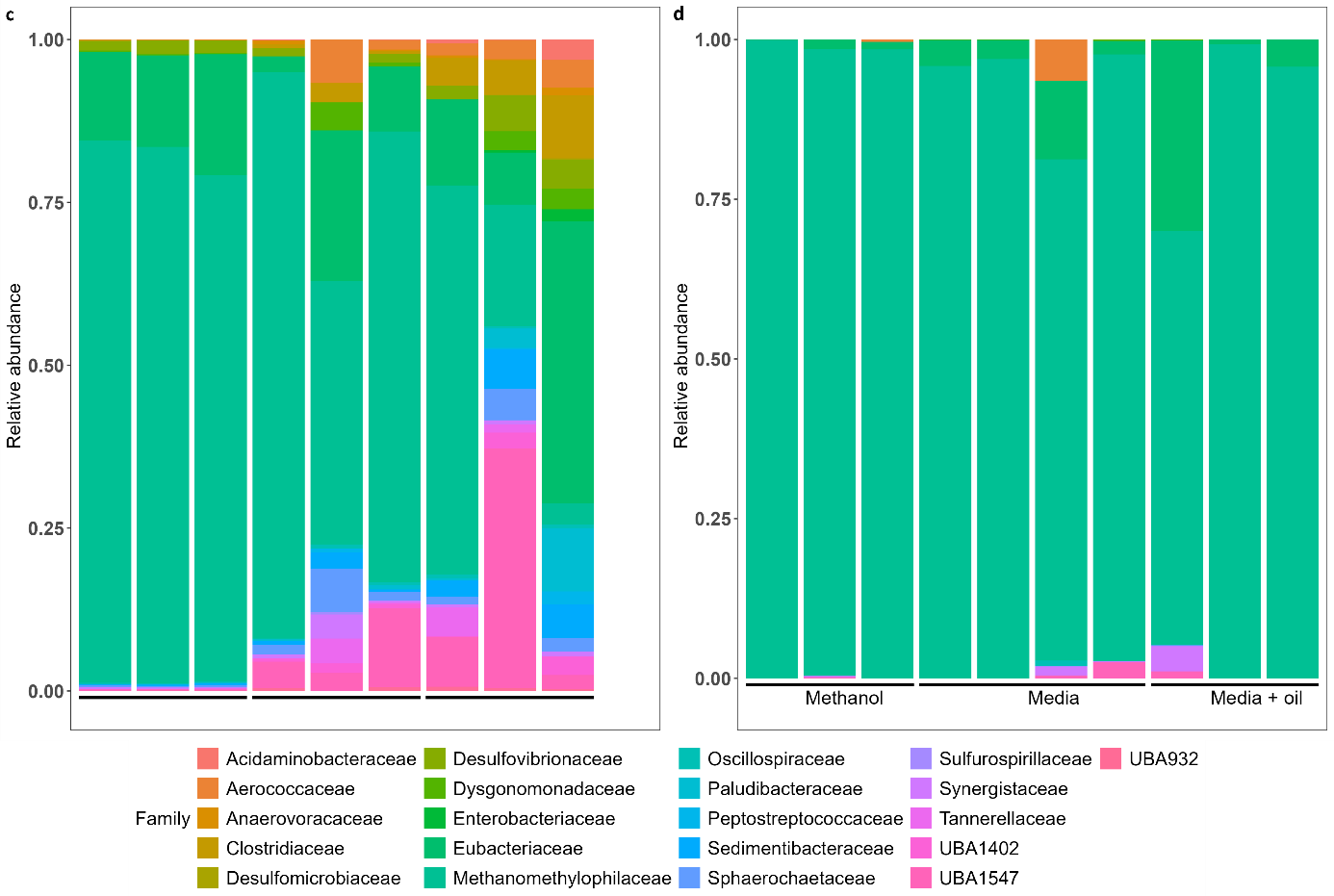
**

**
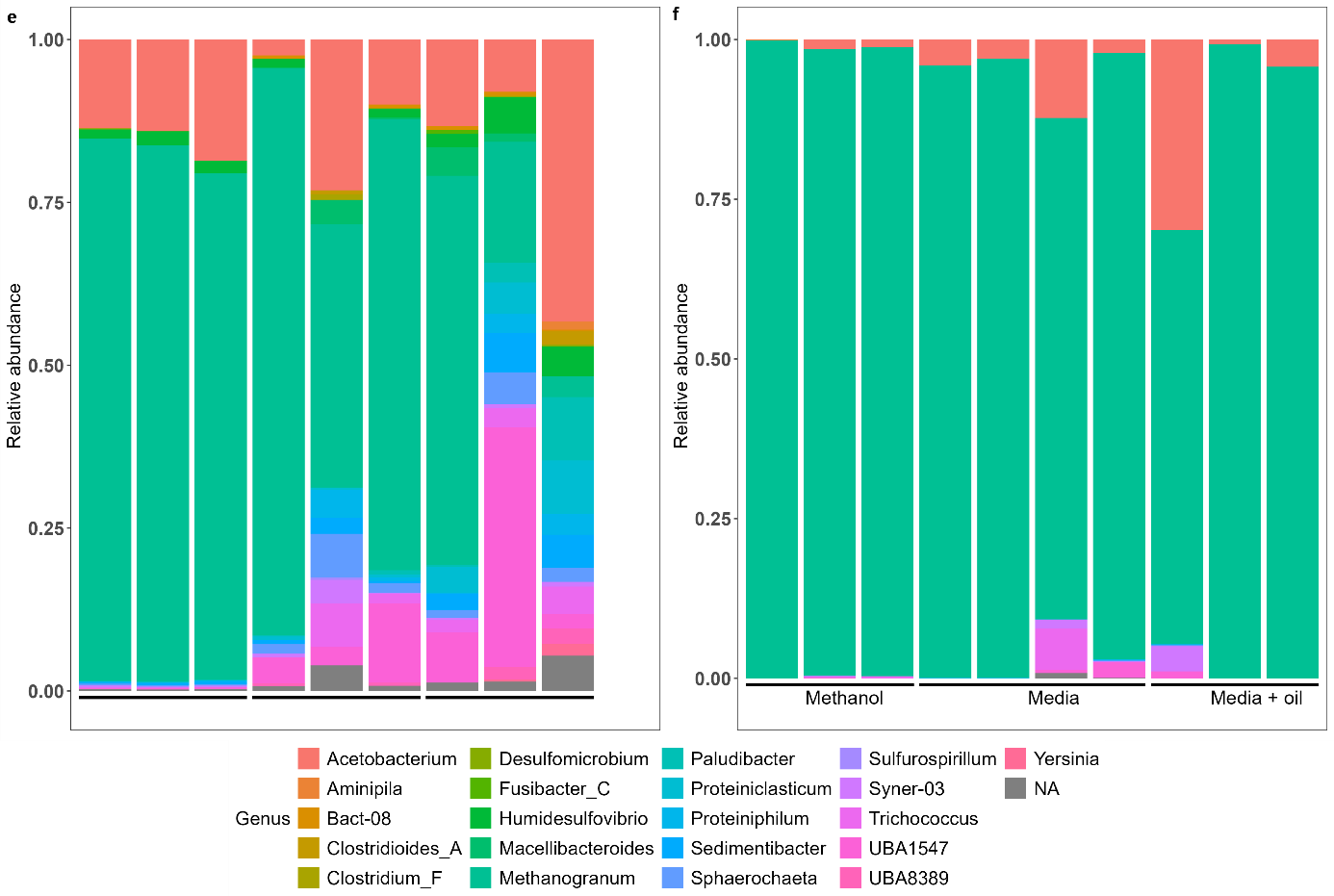
**

**Supplementary Figure 7. Species richness across treatments.** Chao1 species richness were compared across treatments M, MO and MM. The estimated species richness show treatment-specific differences, though no significant difference (*p* = 0.058, Kruskal-Wallis).

**
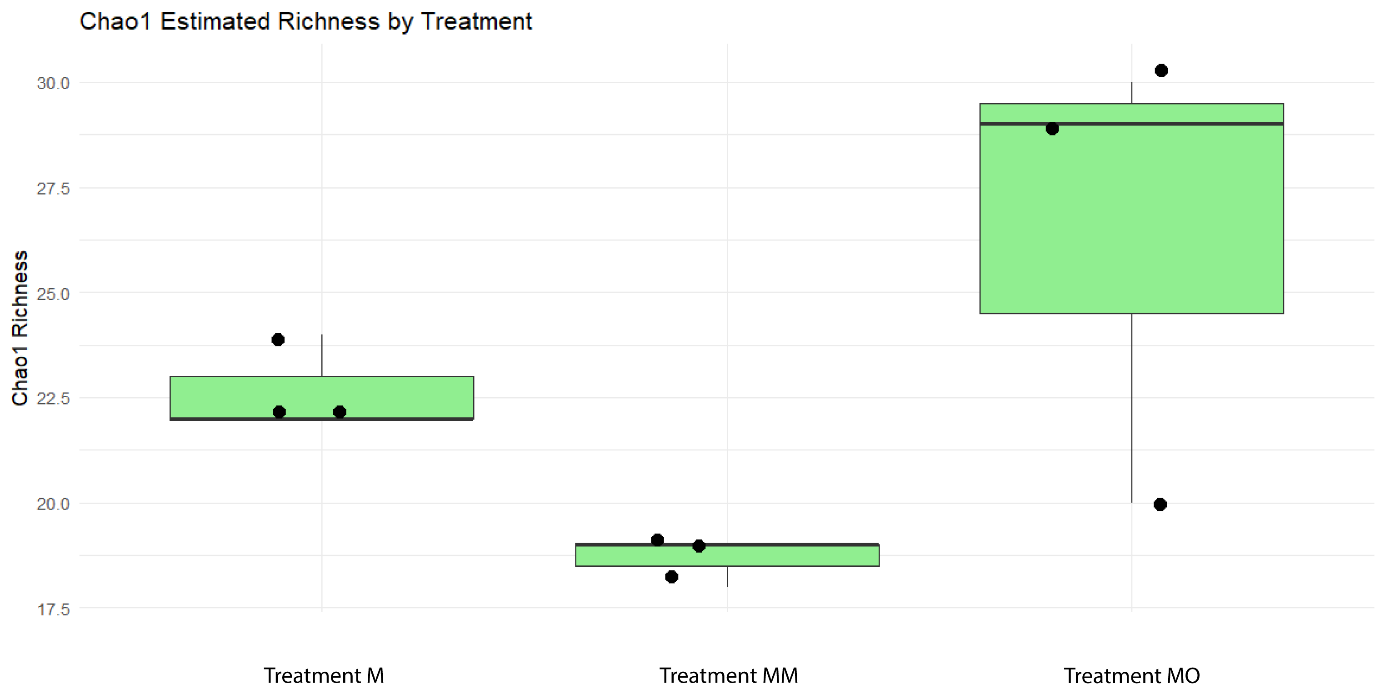
**

**Supplementary Figure 8. RNA transcript-based active genes.** Genes were assiged to taxa based on the identified Metabolic_C functions in Figure 4 and Kegg Orthologs (Supplemental Data 4).

**
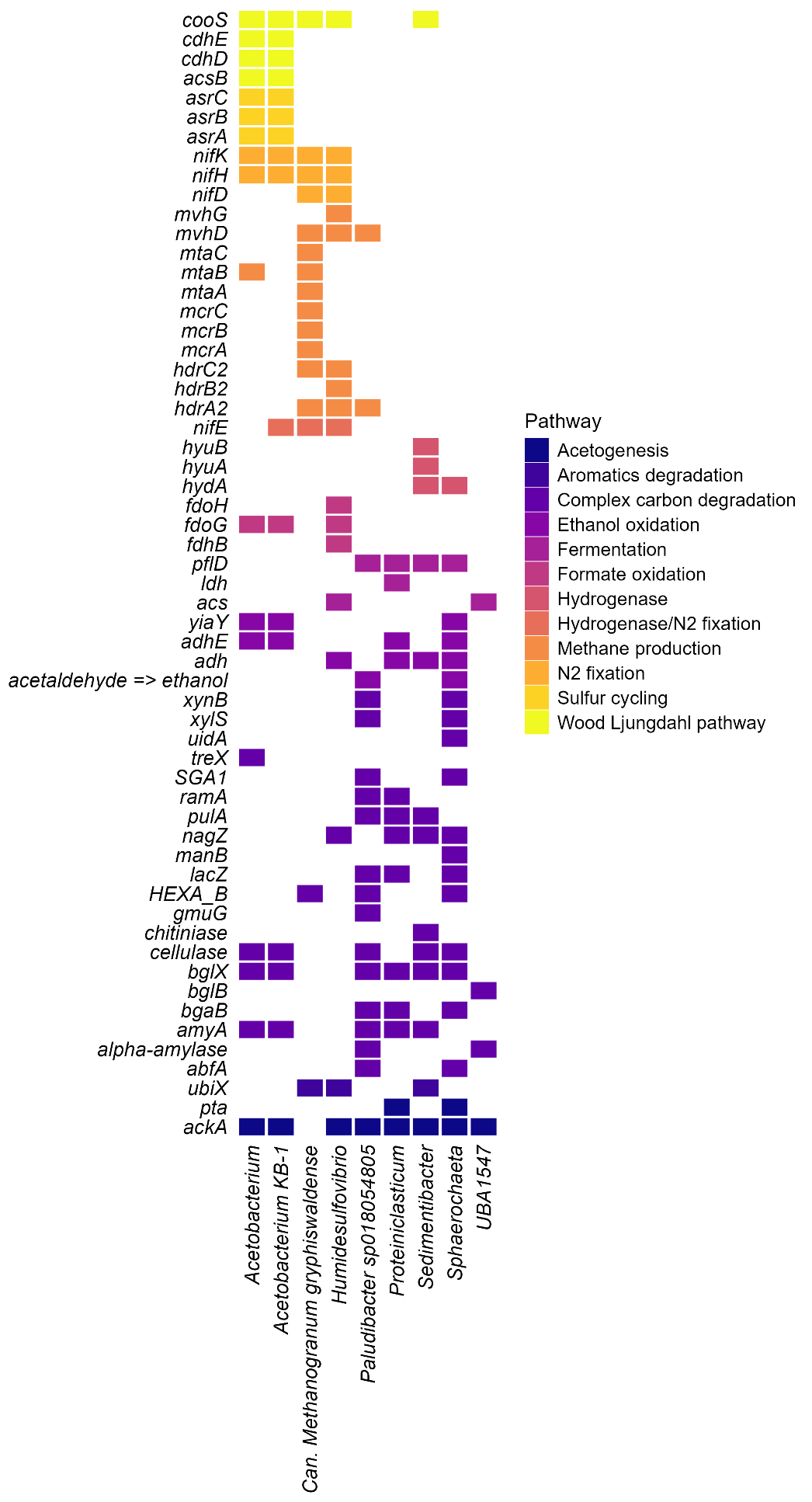
**

**Supplementary references**

1. Starr EP, Shi S, Blazewicz SJ *et al.* Stable isotope informed genome-resolved metagenomics reveals that Saccharibacteria utilize microbially-processed plant-derived carbon. *Microbiome* 2018;**6**:122.

2. Figueroa-Gonzalez PA, Bornemann TL V., Adam PS *et al.* Saccharibacteria as Organic Carbon Sinks in Hydrocarbon-Fueled Communities. *Front Microbiol* 2020;**11**, DOI: 10.3389/fmicb.2020.587782.

3. Révész F, Figueroa-Gonzalez PA, Probst AJ *et al.* Microaerobic conditions caused the overwhelming dominance of *Acinetobacter* spp. and the marginalization of Rhodococcus spp. in diesel fuel/crude oil mixture-amended enrichment cultures. *Arch Microbiol* 2020;**202**:329–42.

4. Salam LB, Ilori MO, Amund OO *et al.* Characterization of bacterial community structure in a hydrocarbon-contaminated tropical African soil. *Environ Technol* 2018;**39**:939–51.

5. Luo C, Xie S, Sun W *et al.* Identification of a novel toluene-degrading bacterium from the candidate phylum TM7, as determined by DNA stable isotope probing. *Appl Environ Microbiol* 2009;**75**:4644–7.

6. Rhim JH, Ono S. Combined carbon, hydrogen, and clumped isotope fractionations reveal differential reversibility of hydrogenotrophic methanogenesis in laboratory cultures. *Geochim Cosmochim Acta* 2022;**335**:383–99.

7. Douglas PMJ, Stolper DA, Smith DA *et al.* Diverse origins of Arctic and Subarctic methane point source emissions identified with multiply-substituted isotopologues. *Geochim Cosmochim Acta* 2016;**188**:163–88.

8. Young ED, Kohl IE, Lollar BS *et al.* The relative abundances of resolved ^12^CH_2_D_2_ and ^13^CH_3_D and mechanisms controlling isotopic bond ordering in abiotic and biotic methane gases. *Geochim Cosmochim Acta* 2017;**203**:235–64.

9. Kim JH, Martini AM, Ono S *et al.* Clumped and conventional isotopes of natural gas reveal basin burial, denudation, and biodegradation history. *Geochim Cosmochim Acta* 2023;**361**:133–51.
